# Performance of Physician Groups and Hospitals Participating in Bundled Payments Among Medicare Beneficiaries

**DOI:** 10.1001/jamahealthforum.2022.4889

**Published:** 2022-12-29

**Authors:** Joshua M. Liao, Qian Huang, Erkuan Wang, Kristin Linn, Torrey Shirk, Jingsan Zhu, Deborah Cousins, Amol S. Navathe

**Affiliations:** 1 Department of Medicine, University of Washington School of Medicine, Seattle; 2 Leonard Davis Institute of Health Economics, University of Pennsylvania, Philadelphia; 3 Department of Medical Ethics and Health Policy, Perelman School of Medicine, University of Pennsylvania, Philadelphia; 4 Department of Biostatistics, Perelman School of Medicine, University of Pennsylvania, Philadelphia; 5 Corporal Michael J. Crescenz Veterans Affairs Medical Center, Philadelphia, Pennsylvania

## Abstract

**Importance:**

Hospital participation in bundled payment initiatives has been associated with financial savings and stable quality of care. However, how physician group practices (PGPs) perform in bundled payments compared with hospitals remains unknown.

**Objectives:**

To evaluate the association of PGP participation in the Bundled Payments for Care Improvement (BPCI) initiative with episode outcomes and to compare these with outcomes for participating hospitals.

**Design, Settings, and Participants:**

This cohort study with a difference-in-differences analysis used 2011 to 2018 Medicare claims data to compare the association of BPCI participation with episode outcomes for PGPs vs hospitals providing medical and surgical care to Medicare beneficiaries. Data analyses were conducted from January 1, 2020, to May 31, 2022.

**Exposures:**

Hospitalization for any of the 10 highest-volume episodes (5 medical and 5 surgical) included in the BPCI initiative for Medicare patients of participating PGPs and hospitals.

**Main Outcomes and Measures:**

The primary outcome was 90-day total episode spending. Secondary outcomes were 90-day readmissions and mortality.

**Results:**

The total sample comprised data from 1 288 781 Medicare beneficiaries, of whom 696 710 (mean [SD] age, 76.2 [10.8] years; 432 429 [59.7%] women; 619 655 [85.5%] White individuals) received care through 379 BPCI-participating hospitals and 1441 propensity-matched non−BPCI-participating hospitals, and 592 071 (mean [SD] age, 75.4 [10.9] years; 527 574 [86.6%] women; 360 835 [59.3%] White individuals) received care from 6405 physicians in BPCI-participating PGPs and 24 758 propensity-matched physicians in non−BPCI-participating PGPs. For PGPs, BPCI participation was associated with greater reductions in episode spending for surgical (difference, –$1368; 95% CI, –$1648 to –$1088) but not for medical episodes (difference, –$101; 95% CI, –$410 to $206). Hospital participation in BPCI was associated with greater reductions in episode spending for both surgical (–$1010; 95% CI, –$1345 to –$675) and medical (–$763; 95% CI, –$1139 to –$386) episodes.

**Conclusions and Relevance:**

This cohort study and difference-in-differences analysis of PGPs and hospital participation in BPCI found that bundled payments were associated with cost savings for surgical episodes for PGPs, and savings for both surgical and medical episodes for hospitals. Policy makers should consider the comparative performance of participant types when designing and evaluating bundled payment models.

## Key Points

**Question:**

Do physician group practices participating in bundled payments among Medicare beneficiaries exhibit similar or different changes in episode outcomes compared with participating hospitals?

**Findings:**

This cohort study with a difference-in-differences analysis found that physician group practices participating in bundled payments had associated savings with surgical but not medical episodes, whereas participating hospitals had savings associated with both episode types.

**Meaning:**

The findings of this cohort study suggest that policy makers should consider the comparative performance of participant type when designing and evaluating future bundled payment models.

## Teaser

This cohort study evaluates the association between bundled payments and episode outcomes per medical and surgical episodes for physician groups compared with hospitals participating in the Medicare bundled payments initiative.

## Introduction

Health care policy makers and payers are working to improve results under alternative payment models, in part by determining which organization types to engage in episode-based bundled payments.^1,2^ This effort requires an understanding of how patient outcomes are affected when patients receive care under different participant types, such as hospitals and physician group practices (PGPs).

Although hospital participation in bundled-payment programs has been well studied,^3,4,5,6,7,8,9^ little is known about how PGP participation affects outcomes, particularly compared with hospital participation. Along with hospitals, PGPs participated in model 2 of Medicare’s Bundled Payments for Care Improvement (BPCI) initiative,^10^ assuming accountability for the quality and costs of medical and surgical episodes spanning hospital admission and up to 90 days of postacute care. Although PGP participation in BPCI was associated with reduced Medicare payments and improved quality for joint replacement episodes,^11^ data are lacking for the hundreds of groups participating in other medical and surgical episodes.

To coordinate participation in future payment models, policy makers must understand the dynamics of PGP vs hospital performance, particularly given the evidence from other payment models indicating that physician groups may perform differently than hospitals in managing quality and costs.^12^ We addressed this knowledge gap by evaluating the association between PGPs and hospitals participating in BPCI model 2 on episode outcomes and comparing PGP vs hospital performance.

## Methods

This study was approved by the University of Pennsylvania Institutional Review Board and informed consent was waived because only historical data with minimal risk of harm were used. This study followed the Strengthening the Reporting of Observational Studies in Epidemiology (STROBE) reporting guideline.^13^

### Data Collection

We identified PGPs and hospitals participating in BPCI model 2 and dates of entry by participant-episode from BPCI Initiative Episode Analytic Files from the US Centers for Medicare & Medicaid Services. These data were linked to Provider Enrollment, Chain, and Ownership System files, from which we identified participating physicians’ National Provider Identification numbers from each participating PGP. Using a 20% random national sample of 2011 to 2018 Medicare claims data, we identified patients receiving care through PGPs or hospitals participating in BPCI model 2 for 1 of 10 episodes of interest, as well as patients receiving care through nonparticipating PGPs and hospitals.

Data from the 2011 to 2012 American Hospital Association Annual Survey were used to capture hospital characteristics, and 2011 to 2017 Medicare Provider of Service, Beneficiary Summary, American Community Survey, and Accountable Care Organization files were used to obtain market and additional hospital characteristics. Data from the Medicare Data on Provider Practice and Specialty file,^14^ Physician Compare, and Compendium of US Health Systems^15^ were used to obtain physician characteristics.

### Study Periods, Sample, and Episode Construction

The study period spanned from January 1, 2011, through December 31, 2017, and encompassed a baseline period prior to the start of BPCI (January 1, 2011-September 30, 2013) and a subsequent intervention period (October 1, 2013-December 31, 2017). The study sample included Medicare fee-for-service beneficiaries receiving care through BPCI PGPs and hospitals for 1 of 10 episodes, each defined by a set of Medicare Severity-Diagnosis Related Group codes: the top 5 highest-volume medical episodes (congestive heart failure, pneumonia, sepsis, chronic obstructive pulmonary disease, and other respiratory) and the top 5 highest-volume surgical episodes (lower extremity joint replacement, hip and femur procedures except major joint, percutaneous coronary intervention, upper extremity joint replacement, and spinal fusion). We excluded beneficiaries with end-stage kidney disease or insurance coverage through Medicare Advantage, as well as beneficiaries who had any non-Inpatient Prospective Payment System claims, lacked continuous primary Medicare fee-for-service coverage during or in the 12 months preceding the episode, or died during the index hospital admission.

We constructed episodes beginning with hospital admission and spanning 90 days after hospital discharge, capturing episodes beginning on or before September 30, 2017. To avoid bias arising from Medicare precedence rules for overlapping episodes between participating PGPs and hospitals, we followed prior methods and constructed naturally occurring episodes by assigning overlapping ones to the earlier hospitalization.^3^ We excluded episodes between January 1, 2013, and September 30, 2013, to account for the transitional period during which PGPs and hospitals may have implemented care process changes in anticipation of BPCI.

### Study Groups

We used Medicare claims data (2011-2018) to identify BPCI PGPs and BPCI hospitals. Groups and hospitals that never participated in any of the 48 episodes in BPCI were categorized as non-BPCI PGPs and non-BPCI hospitals. We used propensity scores to match BPCI with non-BPCI PGPs and BPCI with non-BPCI hospitals (eMethods 1 in Supplement 1). Markets were defined using hospital referral regions^12^ and those with any BPCI PGPs or BPCI hospitals were defined as BPCI Markets. We categorized patients based on hospitalization for 1 of 10 episodes of interest through BPCI PGPs, non-BPCI PGPs, BPCI hospitals, or non-BPCI hospitals. Patients receiving care through BPCI PGPs and hospitals were categorized as “BPCI-both.”

### Exposure and Covariates

The study exposures were dichotomous indicators of PGP participation and hospital participation in BPCI. To reflect participant entry into BPCI at different times, participation indicators were time-varying and specific to each PGP or hospital. Groups and hospitals were considered as participants after enrolling in BPCI, regardless of subsequently dropping out. Covariates were chosen based on prior studies and included patient demographic and clinical variables, such as age, sex, and disease severity (defined using Elixhauser comorbidities), as well time-varying market variables, such as Medicare population size and Medicare Advantage penetration.^3,4,16,17^ Race and ethnicity data were obtained from Medicare claims data, which included Black and White race categories that were used directly in the analyses; however, Asian, Hispanic, Native American (including American Indian, Alaska Native, Native Hawaiian, or other Pacific Islander), and other responses were classified in the “other” category.

### Study Outcomes

The study’s primary outcome was 90-day total episode spending (actual spending adjusted for inflation to 2018 US dollars). Secondary outcomes were 90-day readmissions and 90-day mortality. We also evaluated a number of exploratory spending and utilization outcomes (eMethods 2 in Supplement 1).

### Statistical Analysis

Characteristics between the propensity-matched BPCI and non-BPCI PGPs and BPCI and non-BPCI hospitals were compared using standardized differences of means and proportions.^18^ We used a difference-in-differences (DID) method to mimic a 2 × 2 factorial experiment comparing BPCI participation among hospitals and PGPs. Per the factorial design, we classified the treatment groups to reflect patient exposure to BPCI hospitals, BPCI PGPs, both (BPCI-both), and neither (non-BPCI). This approach enabled the comparison of episode performance for BPCI PGPs (vs non-BPCI) and BPCI hospitals (vs non-BPCI), as well as between BPCI PGPs and BPCI hospitals).

In adjusted analyses, DID models included PGP- and hospital-specific indicators of BPCI participation as treatment to reflect the time-varying nature of BPCI participation—that is, the fact that PGPs and hospitals could start participating at different times. This approach contrasted with traditional DID models, in which the baseline and treatment periods are fixed regardless of timing of actual contract initiation.^19^ Nonparticipating PGPs and hospitals were assigned the same treatment indicators as their propensity matched organizations. We evaluated medical and surgical episodes separately because they involve different care processes that may have different associations with outcomes.^3,5,19^

We used generalized linear models with identity links and normal distributions for all outcomes. All models included episode (Medicare Severity-Diagnosis Related Group code) and market (hospital referral regions) fixed effects to generate within-episode type, within-market estimates that addressed time-invariant episode type, and geographic differences. Models also included time (calendar quarter) fixed-effects to account for secular trends. Robust standard errors were clustered at the hospital level.

We took steps to examine assumptions of our DID method for the primary outcome. First, we examined but did not observe divergent trends for the primary outcome in the baseline period for study groups (eFigures 1 and 2 in Supplement 1). Second, given concerns regarding time-varying DID designs,^20^ we assessed the relationship between the primary outcome and potentially time-varying market-level covariates and how those covariates changed over time across the 4 study groups (eMethods 2 in Supplement 1). Covariates were deemed potential confounders if their association with an outcome varied over time or if their distributions changed differentially over time for BPCI vs non-BPCI PGPs or hospitals in the propensity matched samples. We identified 3 time-varying confounders: Medicare Advantage penetration, acute care organization penetration, and hospital concentration as measured by the Herfindahl-Hirschman index, and accounted for each by including interaction terms between each and a time variable in models. Third, given the potential for time-varying patient selection based on factors that were unobservable in study data, we assessed but did not observe changes in the association between patient Elixhauser comorbidity score and the primary outcome over time across study groups.

Final model specifications for the primary outcome included only covariates deemed as potential confounders according to this process (eMethods 3 in Supplement 1). The Holm−Bonferroni method was used to correct for multiple testing across study groups for the primary study outcome.^21^ Under this approach, statistical significance was defined for total episode spending as *P* = .0125, .017, .025, and .05 for a ranked set of 4 comparisons. Analyses were conducted from January 1, 2020, to May 31, 2022, using SAS, version 9.4 (SAS Institute).

### Sensitivity and Other Analysis

We accounted for skewness in spending by using nonlinear models with log links and gamma distributions to analyze spending. Additionally, to reflect BPCI initiative precedence rules, we used models that considered patients in the group BPCI-both (those receiving care from both PGP and hospital participants) as being in the BPCI PGP group. Finally, we repeated analyses for the mortality outcome by including individuals who died during index hospitalization.

In response to an association observed between BPCI participation and differential changes in mortality that, to our knowledge, has not been previously described in the literature, we conducted post hoc analyses to explore robustness. First, we described selection based on observable and unobservable patient characteristics. Second, we tested approaches to mitigate bias from unobserved clinical severity of patients. Third, we repeated analyses using our modeling approach, but using data from that earlier time period, to assess the ability to replicate those mortality results.

## Results

The total study sample comprised 2011 to 2018 Medicare claims data for 1 288 781 beneficiaries, of whom 696 710 patients (mean [SD] age, 76.2 [10.8] years; 432 429 [59.7%] women; 54 854 [7.6%] Black, 619 655 [85.5%] White, and 50 348 [7.0%] individuals of other race and ethnicity) received care through 379 BPCI hospitals and 1441 propensity-matched non-BPCI hospitals; and 592 071 patients (mean [SD] age, 75.4 [10.9] years; 360 835 [59.3%] women; 41 660 [6.8%] Black, 527 574 [86.6%] White, and 39 783 [6.5%] individuals of other race and ethnicity) received care from 6405 physicians in BPCI PGPs and 24 758 propensity-matched physicians in non-BPCI PGPs.

### Hospital and Physician Characteristics

Compared with non−BPCI-participating hospitals, participating hospitals tended to be larger, nonprofit, teaching hospitals located in urban areas and markets with larger populations and smaller proportions of low-income individuals (eTable 1 in Supplement 1). Physicians participating in BPCI vs non-BPCI PGPs differed in several ways including that the former tended to be younger, to care for fewer beneficiaries, and to be affiliated with a health system (eTable 2 in Supplement 1). Differences between BPCI-participating and non-BPCI hospitals, as well as BPCI-participating physicians and non-BPCI PGPs, decreased after propensity score matching, with standardized mean differences of 0.1 or less for all variables.

### Medical Episodes

**Figure 1.**
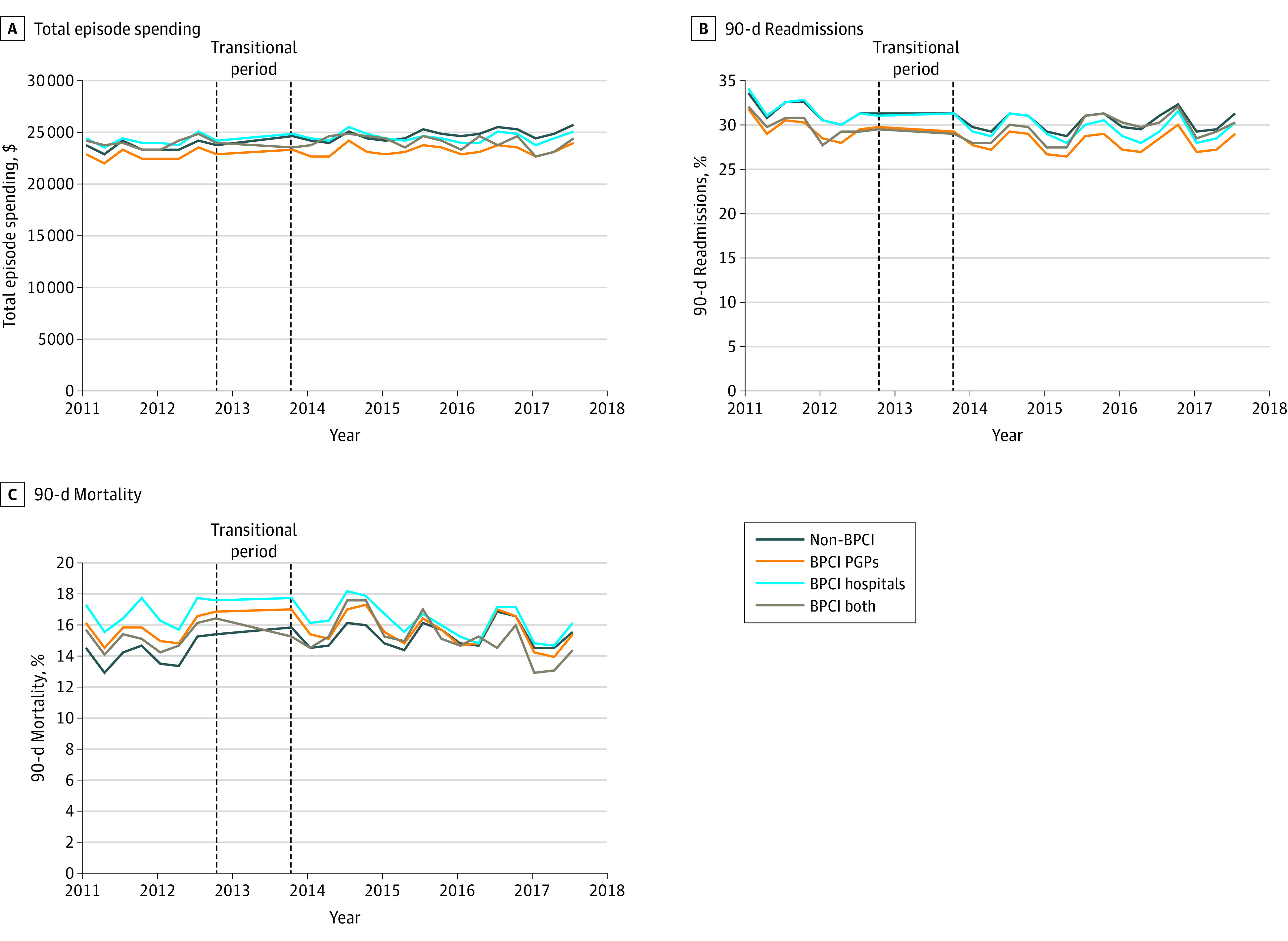
Changes in Total Episode Spending, Readmissions, and Mortality for Medical Episodes, by Study Group A, The preperiod means for total episode spending were: non-BPCI, $23 941; BPCI PGPs, $22 903; BPCI hospitals, $24 389; BPCI both, $23 952. B. The preperiod means for readmissions were: non-BPCI, 30.9%; BPCI PGPs, 28.9%; BPCI hospitals, 30.9%; BPCI both, 29.0%. C, The preperiod means for mortality were: non-BPCI, 14.6%; BPCI PGPs, 15.8%; BPCI hospitals, 16.9%; BPCI both, 15.4%. BPCI indicates Bundled Payments for Care Improvement; PGP, physician group practice.

For medical episodes, total episode spending increased, whereas 90-day readmissions and mortality decreased over time (Figure 1; eFigure 3 in Supplement 1). In the baseline period, some characteristics differed among patient groups receiving care for medical episodes through BPCI PGP, BPCI hospital, BPCI-both, and non-BPCI groups (Table 1). Market characteristics also varied by group in the baseline period for medical episodes (eTable 3 in Supplement 1).

**Table 1.** Characteristics of Patients Admitted for Medical Episodes, by Participation in Bundled Payments, During Baseline Period

Characteristic	Non-BPCI	BPCI PGP	BPCI hospital	BPCI both
Episodes, No.	312 551	44 831	35 172	3994
Patients, No.	299 246	44 470	34 765	3983
Female sex, No. (%)	176766 (56.6)	25252 (56.3)	19929 (56.7)	2206 (55.2)
Male sex, No. (%)	135785 (43.4)	19579 (43.7)	15243 (43.3)	1788 (44.8)
Age, mean (SD), y	76.5 (12.1)	76.2 (12.1)	77.8 (12.0)	77.2 (12.3)
Race, No. (%)				
Black	27 702 (8.9)	3653 (8.2)	3518 (10.0)	326 (8.2)
White	262 328 (83.9)	37 757 (84.2)	28 460 (80.9)	3334 (83.5)
Other^a^	22 521 (7.2)	3421 (7.6)	3194 (9.1)	334 (8.4)
Disabled, No. (%)	42 910 (13.7)	6403 (14.3)	4107 (11.7)	533 (13.4)
Medicare−Medicaid dual eligible, No. (%)	84 864 (27.2)	12 633 (28.2)	9368 (26.6)	1029 (25.8)
Elixhauser comorbidity index, mean (SD), points	20.1 (14.1)	19.5 (14.0)	20.5 (14.0)	20.4 (14.0)
Prior use, mean (SD), %^b^				
IRF	3.4 (18.0)	3.2 (17.6)	3.6 (18.7)	2.9 (16.8)
SNF	19.6 (39.7)	17.1 (37.6)	19.8 (39.9)	18.5 (38.8)
Hospital	52.9 (49.9)	47.6 (49.9)	49.7 (50.0)	45.9 (49.8)
Episodes, No.				
Congestive heart failure	67 322	8387	10 057	968
COPD/bronchitis/asthma	64 925	9967	7093	756
Pneumonia/pleurisy	92 256	14 336	10 749	1321
Respiratory infection/inflammation	30 628	5144	2470	234
Sepsis	57 420	6997	4803	715

Abbreviations: BPCI, Medicare Bundled Payments for Care Improvement initiative; IRF, inpatient rehabilitation facility; COPD, chronic obstructive pulmonary disease, PGP, physician group practice; SNF, skilled nursing facility.

^a^ Includes Asian, Hispanic, Native American (American Indian, Alaska Native, Native Hawaiian, other Pacific Islander), and other responses as reported from the Medicare claims data.

^b^ Use within the previous 12 months.

Between baseline and intervention periods (eTables 5 and 6 in Supplement 1), total episode spending for medical episodes increased in the BPCI PGP and non-BPCI groups and decreased in the BPCI hospital and BPCI-both groups. Study groups differed with respect to unadjusted changes in 90-day readmissions, mortality, and some exploratory outcomes (eTables 5 and 6 in Supplement 1).

In adjusted analysis of medical episodes (eFigure 4A in Supplement 1), patients in the BPCI hospital group had a differential reduction in spending compared with the non-BPCI group (difference, –$763; 95% CI, –$1139 to –$386; *P* < .001). In contrast, there were no differential changes in spending between BPCI PGP and non-BPCI groups (difference, –$102; 95% CI, –$410 to –$206; *P* = .52) or between BPCI-both and non-BPCI groups (difference, –$127; 95% CI, –$875 to –$621; *P* = .74). The BPCI hospitals had differentially greater reductions in total episode spending compared with BPCI PGPs (difference, –$661; 95% CI, –$1118 to –$204; *P* = .005).

Regarding secondary outcomes for medical episodes (eFigure 4B in Supplement 1), there were differential changes in readmissions between BPCI hospital and non-BPCI groups (difference, –0.8 percentage points [pp]; 95% CI, –1.6 to –0.02 pp; *P* = .04), but not between BPCI PGP and non-BPCI groups (difference, –0.1 pp; 95% CI, –0.8 to 0.7 pp; *P* = .83). Compared with the non-BPCI group, there were differential decreases in mortality for BPCI PGPs (difference, –0.7 pp; 95% CI, –1.3 to –0.1 pp; *P* = .02) and BPCI hospitals (difference, –1.1 pp; 95% CI, –1.8 to –0.5 pp; *P* < .001). The BPCI hospitals and BPCI PGPs did not exhibit comparatively differential changes in mortality or readmissions. The BPCI hospitals differed from nonparticipants with respect to exploratory outcomes for medical episodes (eTable 7 in Supplement 1).

### Surgical Episodes

**Figure 2.**
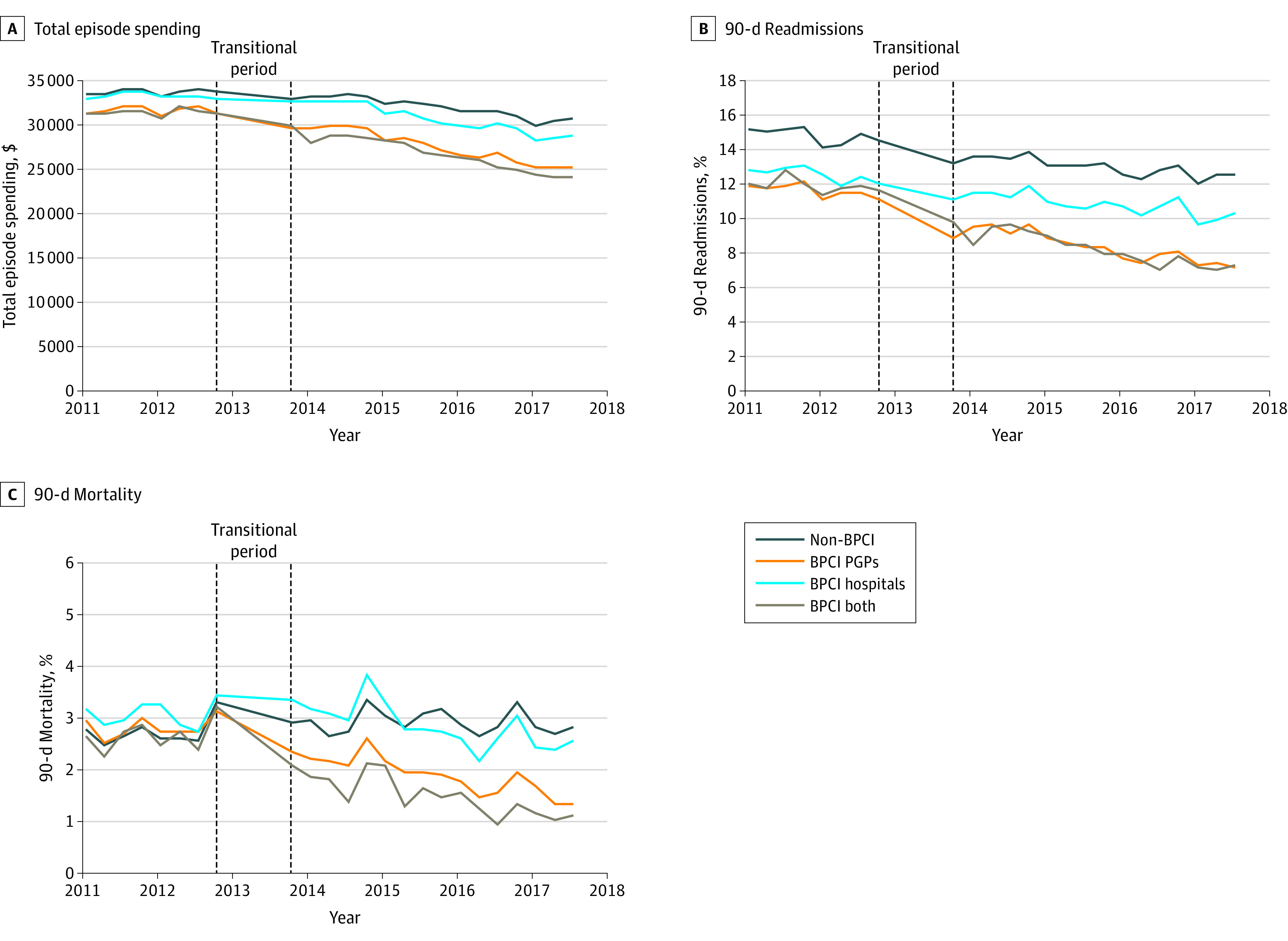
Changes in Total Episode Spending, Readmissions, and Mortality for Surgical Episodes, by Study Group A, The preperiod means for total episode spending were: non-BPCI, $33 557; BPCI PGPs, $30 987; BPCI hospitals, $33 135; BPCI both, $31 055. B, The preperiod means for readmissions were: non-BPCI, 14.2%; BPCI PGPs, 10.7%; BPCI hospitals, 12.1%; BPCI both, 11.2%. C, The preperiod means for mortality were: non-BPCI, 2.8%; BPCI PGP, 2.6%; BPCI hospital, 3.3%; BPCI both, 2.5%. BPCI indicates Bundled Payments for Care Improvement; PGP, physician group practice.

Total episode spending for surgical episodes increased over time, whereas 90-day readmissions and mortality decreased over time (Figure 2; eFigure 5 in Supplement 1). Study groups differed by patient (Table 2) and market characteristics in the baseline period (eTable 4 in Supplement 1). For surgical episodes (eTables 8 and 9 in Supplement 1), total episode spending decreased between the baseline and intervention periods in all groups, with the largest decrease among patients in BPCI-both and the smallest decrease among patients in non-BPCI. Across groups, there were some differences in changes in 90-day readmissions, mortality, and exploratory outcomes (eTable 8 and 9 in Supplement 1).

**Table 2.** Characteristics of Patients Admitted for Surgical Episodes, by Participation in Bundled Payments, During Baseline Period

Characteristic	Non-BPCI	BPCI PGP	BPCI hospital	BPCI both
Episodes, No.	207 716	27 658	35 480	2971
Patients, No.	205 986	27 624	35 446	2970
Female sex, No. (%)	131 081 (63.1)	17 555 (63.5)	22 846 (64.4)	1951 (65.7)
Male sex, No. (%)	76 635 (36.9)	10 103 (36.5)	12 634 (35.6)	1020 (34.3)
Age, mean (SD) y	74.9 (9.6)	74.7 (9.0)	75.1 (9.5)	74.9 (8.7)
Race, No. (%)				
Black	11 166 (5.4)	1293 (4.7)	2170 (6.1)	196 (6.6)
White	184 823 (89.0)	25 057 (90.6)	30 890 (87.1)	2640 (88.9)
Other^a^	11 727 (5.7)	1308 (4.7)	2420 (6.8)	135 (4.5)
Disabled, No. (%)	18 987 (9.1)	2051 (7.4)	2924 (8.2)	194 (6.5)
Medicare/Medicaid dual eligible, No. (%)	27 923 (13.4)	3041 (11.0)	4643 (13.1)	298 (10.0)
Elixhauser comorbidity index, mean (SD), points	7.2 (11.9)	5.8 (11.1)	6.5 (11.4)	5.9 (11.2)
Prior use, mean (SD), %^b^				
IRF	2.2 (14.5)	1.4 (11.6)	1.9 (13.7)	1.5 (12.2)
SNF	7.7 (26.7)	6.1 (23.9)	7.2 (25.8)	7.1 (25.6)
Hospital	29.8 (45.7)	27.5 (44.7)	25.4 (43.6)	24.2 (42.8)
Episodes, No.				
Hip/femur procedure (not major joint)	24 295	3687	3412	219
Lower extremity joint replacement	147 774	19 619	27 782	2662
Percutaneous coronary intervention	17 924	1654	2489	38
Spinal fusion	11 737	1574	1392	43
Upper extremity joint replacement	5986	1124	405	9

Abbreviations: BPCI, Medicare Bundled Payments for Care Improvement initiative; IRF, inpatient rehabilitation facility; PGP, physician group practice; SNF, skilled nursing facility.

^a^ Includes Asian, Hispanic, Native American (American Indian, Alaska Native, Native Hawaiian, other Pacific Islander), and other responses as reported from the Medicare claims data.

^b^ Use within the previous 12 months.

In adjusted analysis (eFigure 6A in Supplement 1), there were differentially greater reductions in total episode spending for surgical episodes for the BPCI hospital group (difference, –$1010 vs non-BPCI; 95% CI, –$1345 to –$675; *P* < .001), BPCI PGP group (difference, –$1345 vs non-BPCI; 95% CI, –$1674 to –$1016; *P* < .001), and BPCI-both (difference, –$1584; 95% CI, –$2326 to –$843; *P* < .001) compared with the non-BPCI group. The magnitude of spending changes did not differ between BPCI hospitals and PGPs (difference, $335; 95% CI, –$97 to $767; *P* = .13).

Compared with patients in the non-BPCI group, those in the BPCI PGP group had differentially greater changes in 90-day readmissions (–0.6 pp; 95% –1.1 to –0.04 pp; *P* = .04). In contrast, there were no differential changes in readmissions for the BPCI hospital or BPCI-both groups compared with the non-BPCI group (eFigure 6B in Supplement 1). The BPCI hospitals had differential increase in readmissions compared with changes for BPCI PGPs (0.7 pp; 95% CI, 0.01 to 1.4 pp; *P* = .047).

Mortality changed differentially for patients cared for through BPCI PGPs (difference, –0.5 pp; 95% CI, –0.8 to –0.2 pp; *P* = .002) and BPCI hospitals (difference, –0.7 pp; 95% CI, –0.9 to –0.4 pp; *P* < .001) compared with patients in the non-BPCI group. The magnitude of these changes was not different for BPCI hospitals vs BPCI PGPs (difference, –0.2 pp; 95% CI, –0.6 to 0.2 pp; *P* = .26). Compared with nonparticipants, BPCI hospitals and PGPs differed in exploratory surgical episode outcomes (eTable 10 in Supplement 1).

### Sensitivity Analysis

Compared with the main study analyses, results of sensitivity analyses were qualitatively similar (eFigures 7-12 in Supplement 1). Post hoc analyses demonstrated differential reductions in observed and unobserved severity over time for the BPCI hospital and PGP vs non-BPCI groups (eMethods 4 in Supplement 1). Although models to adjust for unobserved severity did not produce differential results compared with original models, these approaches were limited in correlation with patient-level observed mortality, thereby limiting the strength of the conclusions. Analyses using older data ranges replicated findings from previous studies^3^ that found no association between BPCI hospital participation and differential mortality changes.

## Discussion

In this cohort study with DID analysis, participation of PGPs and hospitals in BPCI was associated with cost savings for surgical episodes; however, only hospital participation was associated with cost savings for medical episodes. Hospital and PGP participation were associated with different patterns of changes in postacute utilization and mortality. For example, for medical episodes, hospital participation in BPCI was significantly associated with reductions in length of stay at skilled nursing facilities, whereas PGP participation was not. For surgical episodes, PGP participation in BPCI was associated with reductions in home health use, whereas hospital participation was not. These findings pose 3 implications.

First, these findings underscore the benefit of engaging PGPs in episode-based payment models. This analysis adds to prior work by describing the association of PGP participation in BPCI with cost savings for multiple surgical episodes, extending beyond hip and knee replacements.^11^ Although it is only one aspect of a payment model’s success, spending reductions are critical because policy makers increasingly judge the viability of bundled payment programs by their cost savings.^22^

Second, these study findings affirm the suitability of hospitals to bundled payment models, specifically highlighting their relative advantage over PGPs in achieving cost and potential quality outcomes for medical conditions. These findings contrast with prior research^12^ demonstrating that PGPs may be more successful than hospitals at reducing spending in population-based payment models, such as acute care organizations. Future work should elucidate drivers underlying this distinction. For example, hospitals may be better positioned to coordinate with postacute care organizations such as skilled nursing facilities given their high volume of shared patients. These strategies may be particularly important for medical conditions where the episode cost savings come from reductions in postacute care facility length of stay rather than reductions in the proportion of individuals discharged.^3^ Policy makers may consider these facets of performance when considering participant types in future alternative payment models.

Third, these study findings emphasize the need for future research on the drivers of cost savings and quality improvements under bundled payments. Our results regarding BPCI-participating PGPs point to the importance of changes in readmissions and postacute care utilization in determining episode savings. Yet as observed from BPCI-participating hospitals, different patterns of utilization changes may drive savings for different episode types. Specifically, in these findings medical episode savings were associated with reductions in length of stay within skilled nursing facilities, whereas surgical episode cost savings came from fewer discharges to skilled nursing facilities.

Additional work is also needed to assess the relationship between bundled payments and quality improvements. Although differential mortality reductions were observed by this study, there was also evidence of observable and unobservable favorable patient selection under bundled payments. This makes definitive conclusions regarding changes in health care quality challenging. Clarity on whether apparent quality changes represent true improvements, patient selection, or measures of both is highly relevant to policy and should be the focus of future studies.

### Limitations

This study had some limitations worth noting. Findings may have been subject to residual confounding; however, we mitigated concerns by using a DID design that accounted for unobserved heterogeneity and patient and hospital characteristics. We evaluated the highest-volume episodes under a single program; however, BPCI model 2 was the direct basis for ongoing PGP and hospital participation in BPCI Advanced. Also, we did not include more recent data from BPCI Advanced because physician group participation files were not available. Moreover, we were unable to match physicians or identify episodes using tax identification number-level information owing to a lack of data availability. However, our approach using all episodes per National Provider Identification number and any participation in BPCI was conservatively biased toward the null hypothesis. Furthermore, to our knowledge, mortality reductions have not been previously described, and although we tested the robustness of the findings using a range of sensitivity and post hoc analyses, the analyses suggested the presence of unobservable patient selection that precluded definitive conclusions regarding any changes in health care quality. Despite conducting sensitivity analyses using alternative modeling approaches for episode spending as the primary outcome, future work should assess the use of modeling approaches beyond ordinary least-squares for other outcomes.

## Conclusions

This cohort study with DID analysis found that PGP participation in BPCI was associated with cost savings for surgical episodes but not for medical episodes, whereas hospital participation in BPCI was associated with savings for both episode types. Policy makers should consider the comparative performance of participant type when designing and evaluating future bundled payment models.

## Notes

Supplement 1. **eMethods 1.** Propensity Score Matching Approach **eMethods 2.** Exploratory Study Outcomes **eFigure 1.** Adjusted Parallel Trends for Medical Episodes **eFigure 2. **Adjusted Parallel Trends for Surgical Episodes **eMethods 2.** Testing for Time-Varying Confounders in Difference-in-Difference Design **eMethods 3.** Model Specifications **eTable 1.** Characteristics of BPCI and Non-BPCI Hospitals, Before and After Propensity Score Matching **eTable 2.** Characteristics of Physicians in BPCI and Non-BPCI PGPs, Before and After Propensity Score Matching **eFigure 3.** Unadjusted Changes in Medical Episode Outcomes, 2011-2017 **eTable 3.** Characteristics of Medical Episode Patients and Markets, by Study Period and Bundled Payment Status **eTable 4.** Characteristics of Surgical Episode Patients and Markets, by Study Period and Bundled Payment Status **eTable 5.** Unadjusted Changes in Medical Episode Outcomes, Baseline versus Intervention Period **eTable 6.** Percent Changes in Medical Episode Outcomes, Baseline versus Intervention Period **eFigure 4.** Adjusted Changes in Medical Episode Outcomes Associated with Bundled Payment Participation Among PGPs and Hospitals **eTable 7.** Adjusted Changes in Medical Episode Exploratory Outcomes Associated With Bundled Payments **eFigure 5.** Unadjusted Changes in Surgical Episode Outcomes, 2011-2017 **eTable 8.** Unadjusted Changes in Surgical Episode Outcomes, Baseline versus Intervention Period **eTable 9. **Percent Changes in Surgical Episode Outcomes, Baseline versus Intervention Period **eFigure 6.** Adjusted Changes in Surgical Episode Outcomes Associated with Bundled Payment Participation Among PGPs and Hospitals **eTable 10.** Adjusted Changes in Surgical Episode Exploratory Outcomes Associated With Bundled Payments **eFigure 7.** Sensitivity Analysis for Changes in Total Episode Spending in Medical Episodes, Using Generalized Linear Models With Log Link and Gamma Distribution **eFigure 8.** Sensitivity Analysis for Changes in Total Episode Spending in Surgical Episodes, Using Generalized Linear Models With Log Link and Gamma Distribution **eFigure 9.** Sensitivity Analysis for Changes in Total Episode Spending in Medical Episodes, Considering BPCI Both as BPCI PGP episodes **eFigure 10.** Sensitivity Analysis for Changes in Total Episode Spending in Surgical Episodes, Considering BPCI Both as BPCI PGP episodes **eFigure 11.** Sensitivity Analysis for Changes in 90-day Mortality in Medical Episodes, Including Episodes with Death at Index Hospitalization **eFigure 12.** Sensitivity Analysis for Changes in 90-day Mortality in Surgical Episodes, Including Episodes with Death at Index Hospitalization **eMethods 4.** Sensitivity Analysis Examining Robustness of the 90-day Mortality Outcome

Supplement 2.
Data Sharing Statement
