# Supplementary material for: Performance of Physician Groups and Hospitals Participating in Bundled Payments Among Medicare Beneficiaries
Source: JAMA Health Forum. 2022 Dec 29;3(12):e224889. doi: 10.1001/jamahealthforum.2022.4889 (PMC9856773; doi:10.1001/jamahealthforum.2022.4889)
Supplement: Supplement 1. — eMethods 1. Propensity Score Matching Approach eMethods 2. Exploratory Study Outcomes eFigure 1. Adjusted Parallel Trends for Medical Episodes eFigure 2. Adjusted Parallel Trends for Surgical Episodes eMethods 2. Testing for Time-Varying Confounders in Difference-in-Difference Design eMethods 3. Model Specifications eTable 1. Characteristics of BPCI and Non-BPCI Hospitals, Before and After Propensity Score Matching eTable 2. Characteristics of Physicians in BPCI and Non-BPCI PGPs, Before and After Propensity Score Matching eFigure 3. Unadjusted Changes in Medical Episode Outcomes, 2011-2017 eTable 3. Characteristics of Medical Episode Patients and Markets, by Study Period and Bundled Payment Status eTable 4. Characteristics of Surgical Episode Patients and Markets, by Study Period and Bundled Payment Status eTable 5. Unadjusted Changes in Medical Episode Outcomes, Baseline versus Intervention Period eTable 6. Percent Changes in Medical Episode Outcomes, Baseline versus Intervention Period eFigure 4. Adjusted Changes in Medical Episode Outcomes Associated with Bundled Payment Participation Among PGPs and Hospitals eTable 7. Adjusted Changes in Medical Episode Exploratory Outcomes Associated With Bundled Payments eFigure 5. Unadjusted Changes in Surgical Episode Outcomes, 2011-2017 eTable 8. Unadjusted Changes in Surgical Episode Outcomes, Baseline versus Intervention Period eTable 9. Percent Changes in Surgical Episode Outcomes, Baseline versus Intervention Period eFigure 6. Adjusted Changes in Surgical Episode Outcomes Associated with Bundled Payment Participation Among PGPs and Hospitals eTable 10. Adjusted Changes in Surgical Episode Exploratory Outcomes Associated With Bundled Payments eFigure 7. Sensitivity Analysis for Changes in Total Episode Spending in Medical Episodes, Using Generalized Linear Models With Log Link and Gamma Distribution eFigure 8. Sensitivity Analysis for Changes in Total Episode Spending in Surgical Episodes, Using Generalized Linear Model [file jamahealthforum-e224889-s001.pdf]

Supplementary Online Content

Liao JM, Huang Q, Wang E, et al. Performance of physician groups and hospitals participating in bundled payments among Medicare beneficiaries. *JAMA Health Forum*. 2022;3(12):e224889. doi:10.1001/jamahealthforum.2022.4889

eMethods 1. Propensity Score Matching Approach

eMethods 2. Exploratory Study Outcomes

eFigure 1. Adjusted Parallel Trends for Medical Episodes

eFigure 2. Adjusted Parallel Trends for Surgical Episodes

eMethods 2. Testing for Time-Varying Confounders in Difference-in-Difference Design

eMethods 3. Model Specifications

eTable 1. Characteristics of BPCI and Non-BPCI Hospitals, Before and After Propensity Score Matching

eTable 2. Characteristics of Physicians in BPCI and Non-BPCI PGPs, Before and After Propensity Score Matching

eFigure 3. Unadjusted Changes in Medical Episode Outcomes, 2011-2017

eTable 3. Characteristics of Medical Episode Patients and Markets, by Study Period and Bundled Payment Status

eTable 4. Characteristics of Surgical Episode Patients and Markets, by Study Period and Bundled Payment Status

eTable 5. Unadjusted Changes in Medical Episode Outcomes, Baseline versus Intervention Period

eTable 6. Percent Changes in Medical Episode Outcomes, Baseline versus Intervention Period

eFigure 4. Adjusted Changes in Medical Episode Outcomes Associated with Bundled Payment Participation Among PGPs and Hospitals

eTable 7. Adjusted Changes in Medical Episode Exploratory Outcomes Associated With Bundled Payments

eFigure 5. Unadjusted Changes in Surgical Episode Outcomes, 2011-2017

eTable 8. Unadjusted Changes in Surgical Episode Outcomes, Baseline versus Intervention Period

eTable 9. Percent Changes in Surgical Episode Outcomes, Baseline versus Intervention Period

© 2022 Liao JM et al. *JAMA Health Forum.*

eFigure 6. Adjusted Changes in Surgical Episode Outcomes Associated with Bundled Payment Participation Among PGPs and Hospitals
eTable 10. Adjusted Changes in Surgical Episode Exploratory Outcomes Associated With Bundled Payments
eFigure 7. Sensitivity Analysis for Changes in Total Episode Spending in Medical Episodes, Using Generalized Linear Models With Log Link and Gamma Distribution
eFigure 8. Sensitivity Analysis for Changes in Total Episode Spending in Surgical Episodes, Using Generalized Linear Models With Log Link and Gamma Distribution
eFigure 9. Sensitivity Analysis for Changes in Total Episode Spending in Medical Episodes, Considering BPCI Both as BPCI PGP episodes
eFigure 10. Sensitivity Analysis for Changes in Total Episode Spending in Surgical Episodes, Considering BPCI Both as BPCI PGP episodes
eFigure 11. Sensitivity Analysis for Changes in 90-day Mortality in Medical Episodes, Including Episodes with Death at Index Hospitalization
eFigure 12. Sensitivity Analysis for Changes in 90-day Mortality in Surgical Episodes, Including Episodes with Death at Index Hospitalization
eMethods 4. Sensitivity Analysis Examining Robustness of the 90-day Mortality Outcome

This supplemental material has been provided by the authors to give readers additional information about their work.

© 2022 Liao JM et al. *JAMA Health Forum.*

eMethods 1. Propensity Score Matching Approach

We used propensity score matching to decrease differences between BPCI and non-BPCI physician groups and BPCI and non-BPCI hospitals. In particular, we used logistic regression with baseline characteristics (physician and market characteristics for physician group matching; hospital and market characteristics for hospital matching) to estimate a propensity score (value between 0 and 1) for each physician or hospital to be enrolled in BPCI in order to match BPCI and Non-BPCI participant physicians and hospitals. The variables used in propensity score matching are listed in eTables 1 and 2. We used a greedy 3:1 approach and allowed BPCI physicians or hospitals to be matched with 3 non-BPCI physicians or hospitals, respectively[1]. This process achieved good overlap between the BPCI and Non-BPCI groups based on the propensity scores, as demonstrated through the common support graphs below.

We used physician as the primary unit and physician NPI in propensity score matching to maximize consistency between matching and selection into the BPCI treatment exposure. In particular, we used data provided by Medicare – files containing information about physician NPIs associated with BPCI participants; and BPCI enrollment files detailing which BPCI participants enrolled in which episode types – to match NPI and episode type in order to associate physicians across the study period. This approach enabled us to capture physicians participating in BPCI, and their associated episodes, in both the baseline and intervention periods.

Unfortunately, no data were available from Medicare about TINs (or NPI-TIN relationships) associated with BPCI. However, several approaches to conduct matching at the tax identification number (TIN)-level proved infeasible. First, using a TIN-level match to identify non-BPCI physician groups was limited by many-to-many matches occurring between Medicare-provided physician group and TIN units – that is, one TIN could be linked via affiliated physicians with multiple physician groups, and one physician group could be linked via affiliated physicians to multiple TINs. Only 46% of TINs had all physicians belonging to one physician group.

Second, TIN-level matching was also infeasible for identifying both BPCI and non-BPCI physician groups, an approach that would have assigned all physicians to either BPCI or non-BPCI TINs regardless of participation status of individual affiliated physicians. We expected concordance between episodes accounted for by physician groups identified via this approach (via BPCI TINs) versus physician groups reported through program participation data from Medicare – that is, that a large number of episodes captured by BPCI TINs would be verified as BPCI episodes through Medicare program participation data. However, we instead observed a wide range in the proportion of BPCI physician groups within BPCI TINs – that is, BPCI TINs did not necessarily have high large numbers of BPCI physician group episodes. Third, the use of physician-TIN pairs to identify BPCI and non-BPCI physician groups was precluded by few such pairs being present in both the baseline and intervention periods.

© 2022 Liao JM et al. *JAMA Health Forum.*

Common support graphs for BPCI and non-BPCI physician group matching

Congestive Heart Failure	Pneumonia	Sepsis
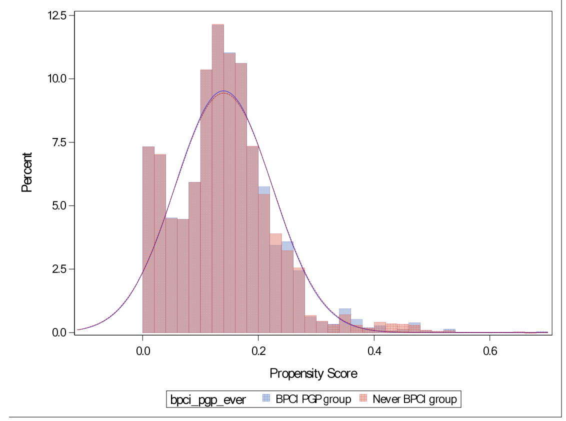	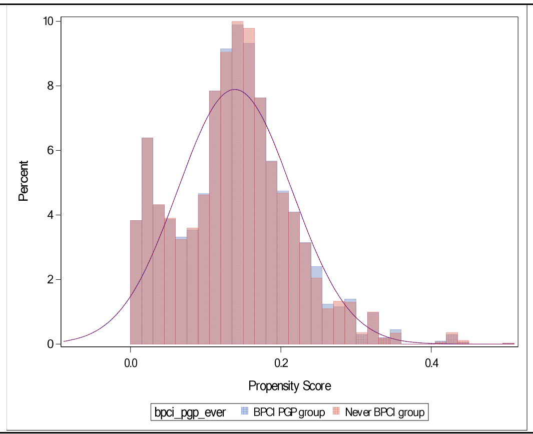	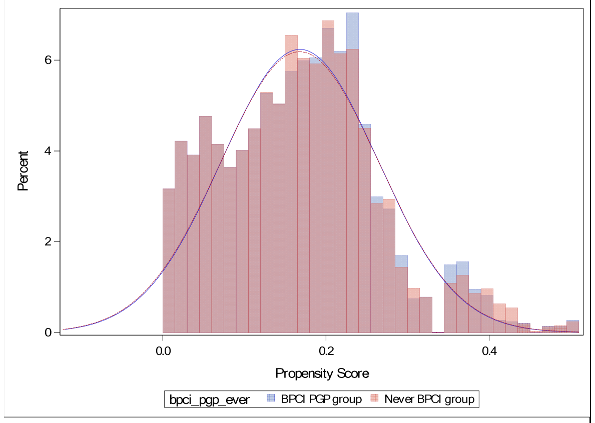
**Chronic Obstructive Pulmonary Disease**	**Other Respiratory**	**Lower Extremity Joint Replacement**
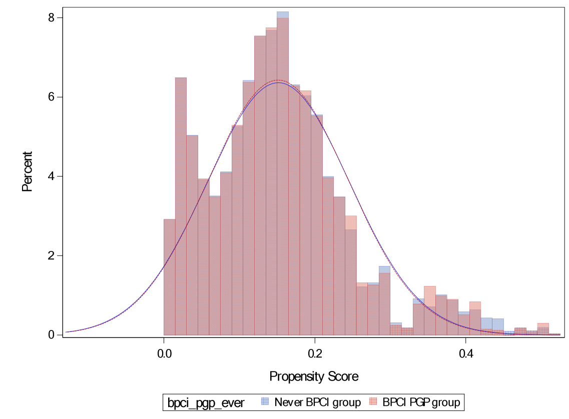	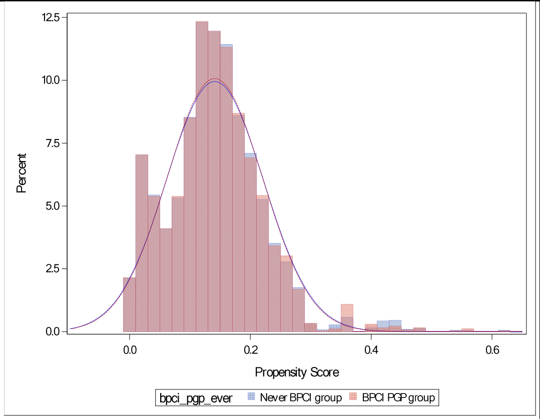	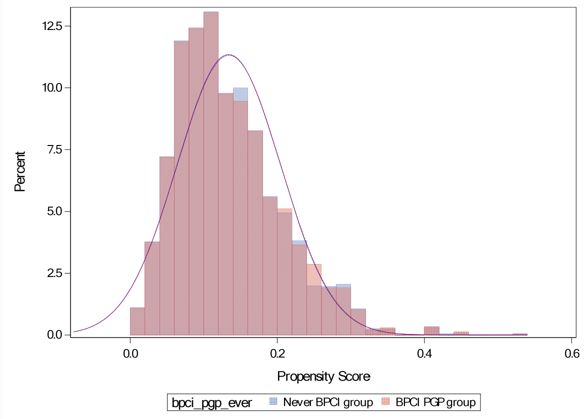
**Hip and Femur Procedures**	**Percutaneous Coronary Intervention**	**Upper Extremity Joint Replacement**

© 2022 Liao JM et al. *JAMA Health Forum.*

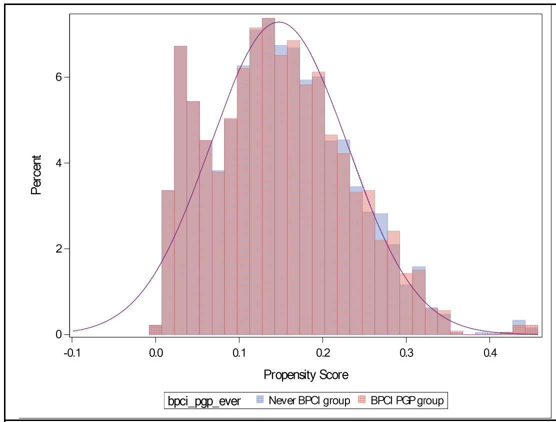

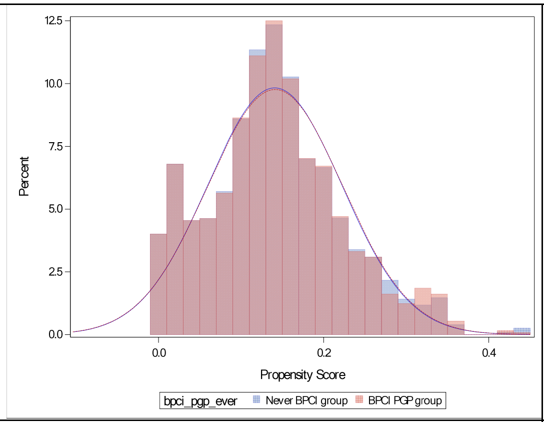

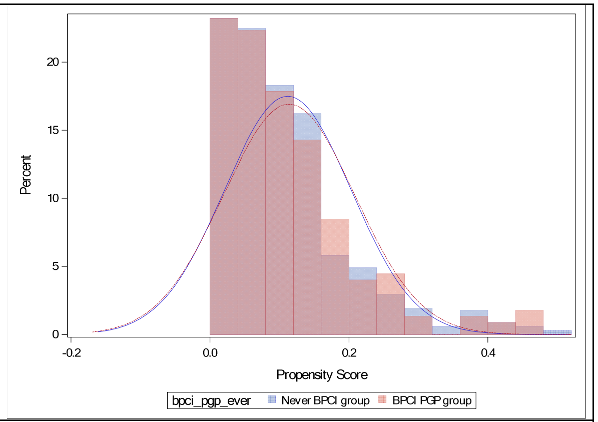

Spinal Fusion

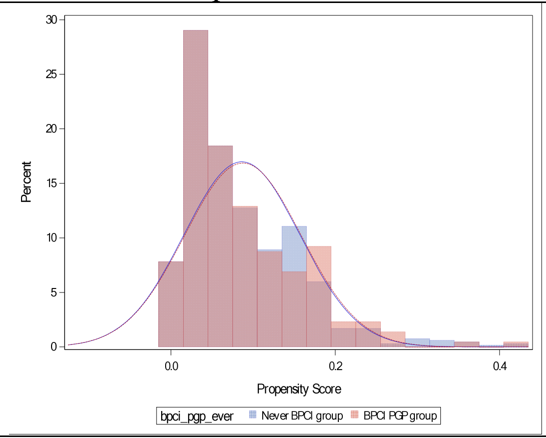

BPCI, Bundled Payments for Care Improvement. PGP = physician group practice.

© 2022 Liao JM et al. *JAMA Health Forum.*

Common support graphs for BPCI and non-BPCI hospital matching

Congestive Heart Failure

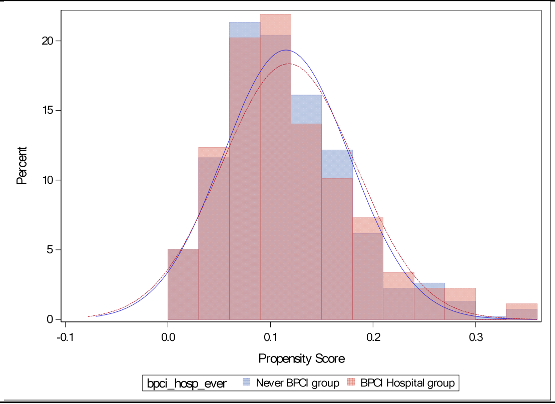

Pneumonia

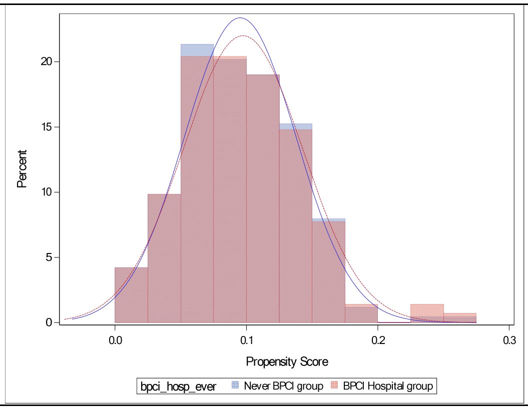

Sepsis

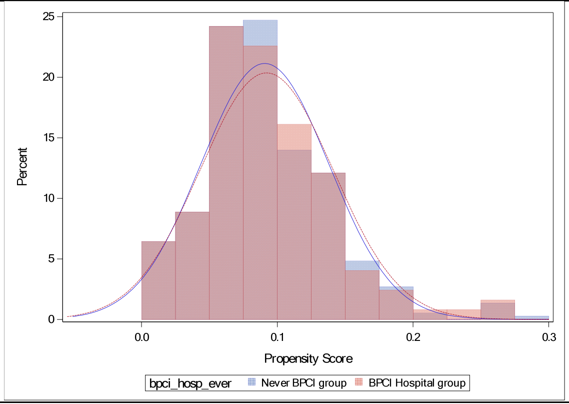

Chronic Obstructive Pulmonary Disease

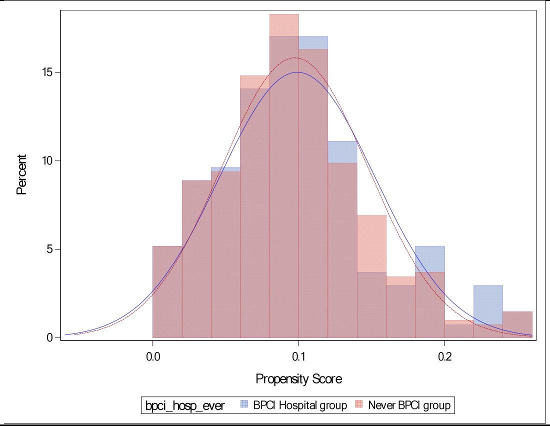

Other Respiratory

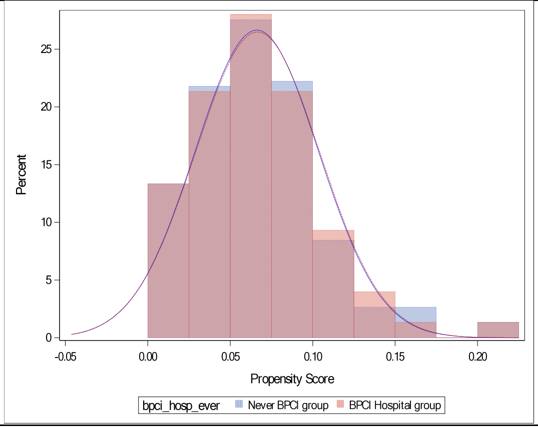

Lower Extremity Joint Replacement

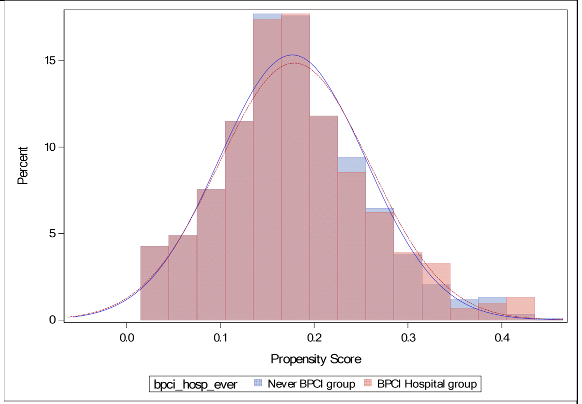

Hip and Femur Procedures

Percutaneous Coronary Intervention

Upper Extremity Joint Replacement

© 2022 Liao JM et al. *JAMA Health Forum.*

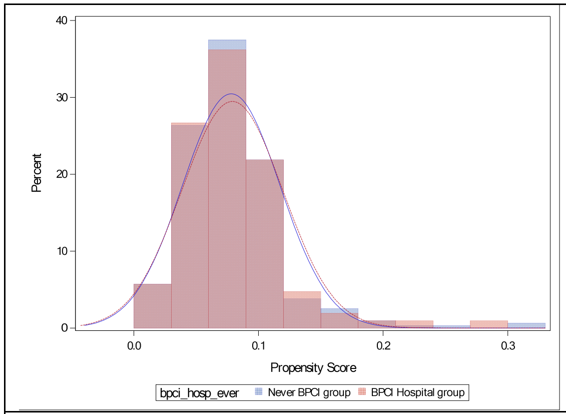

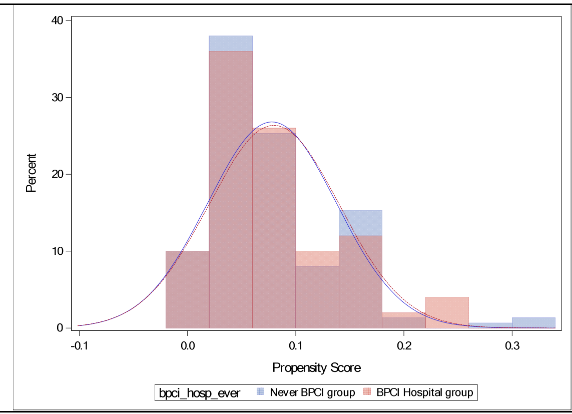

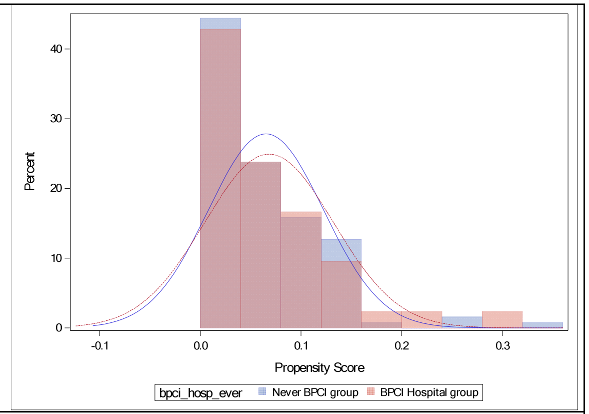

Spinal Fusion

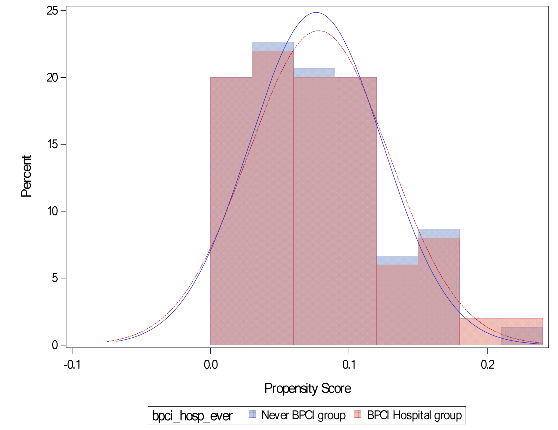

BPCI, Bundled Payments for Care Improvement. PGP = physician group practice.

© 2022 Liao JM et al. *JAMA Health Forum.*

eMethods 2. Exploratory Study Outcomes

Exploratory spending outcomes included the following:

- institutional post-acute care (PAC), defined as the sum of skilled nursing facility (SNF), inpatient rehabilitation facility (IRF) and long-term acute care hospital spending
- home health care agency (HHA) spending

Exploratory utilization outcomes included the following:

- discharge to institutional PAC providers (SNFs or IRFs)
- discharge home with HHA services
- (among patients discharged to SNFs) SNF length of stay (LOS)
- (among patients discharged home with HHA services) HHA days

© 2022 Liao JM et al. *JAMA Health Forum.*

eFigure 1. Adjusted Parallel Trends for Medical Episodes

Although the difference-in-differences assumption of parallel trends involves unobservable counterfactual values and is therefore untestable, we visually examined baseline period data for any suggestion of divergent trends in study outcomes between study groups prior to the start of the BPCI program. For primary and secondary outcomes, we also fit a series of generalized linear regression models in which dependent variables were outcomes and independent variables included time (quarter) fixed effects, a BPCI participant indicator variable (physician group or hospital), their interaction, as well as market (HRR) fixed effects, episode (DRG) fixed effects, and a vector of patient and market characteristics.

Total Episode Spending

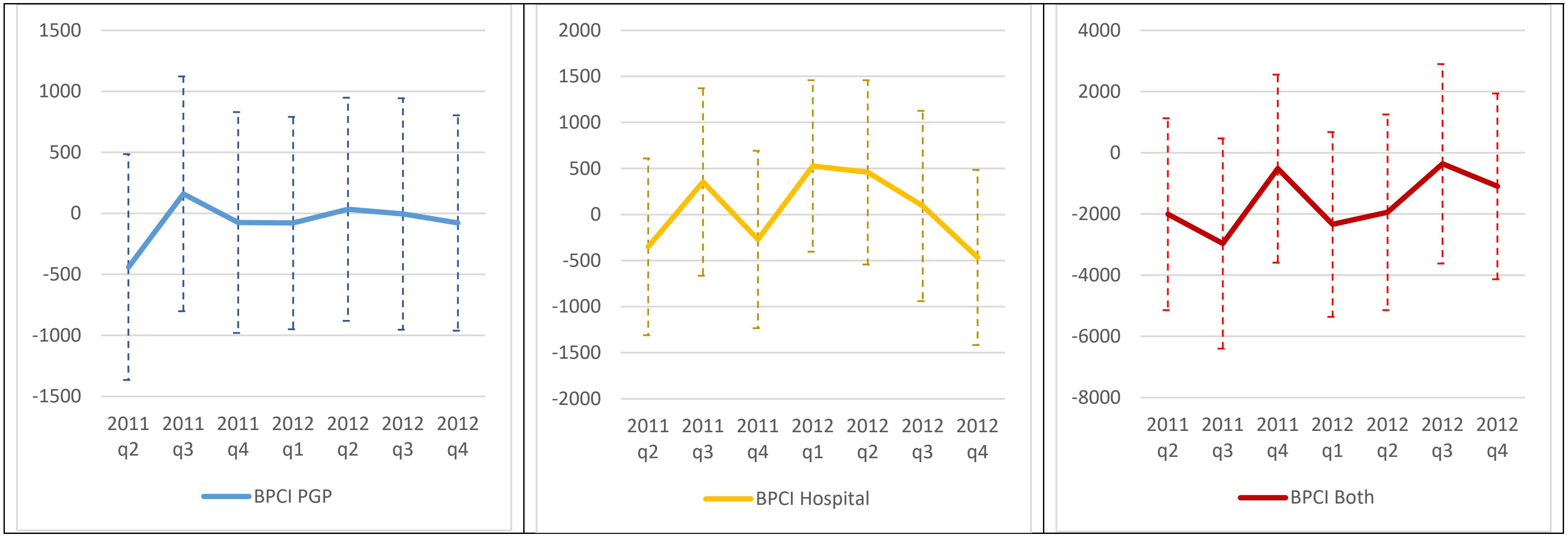

© 2022 Liao JM et al. *JAMA Health Forum.*

90-day Readmissions

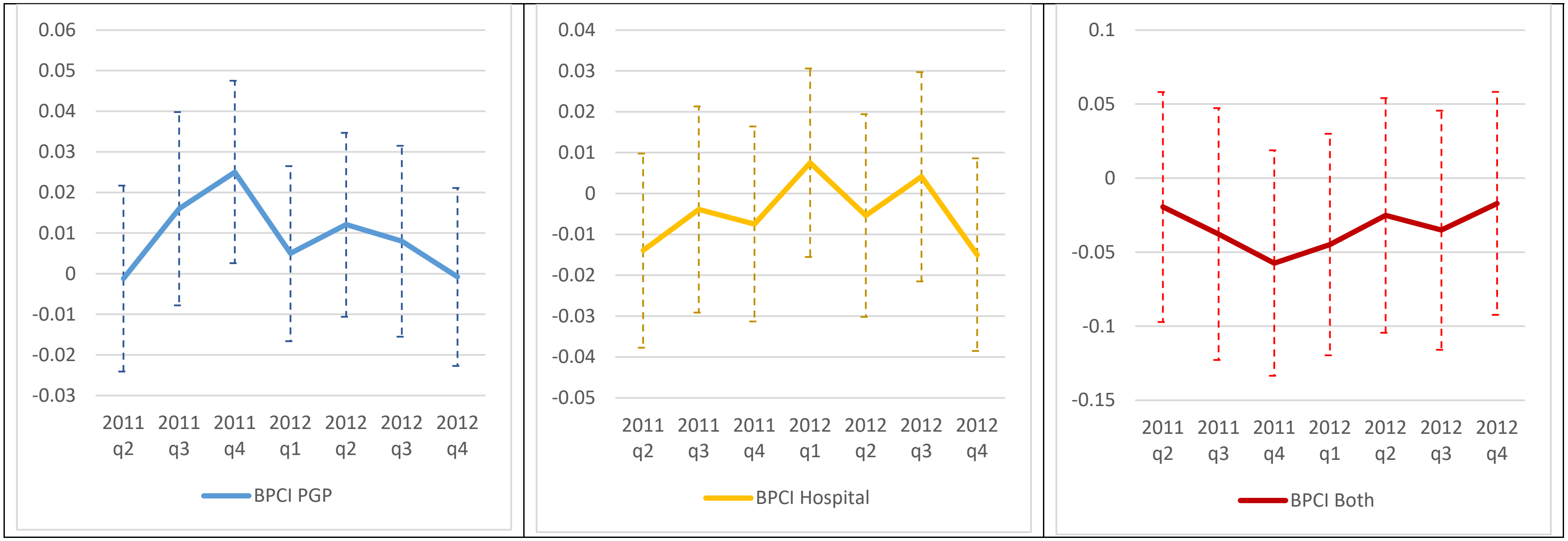

© 2022 Liao JM et al. *JAMA Health Forum.*

90-day Mortality

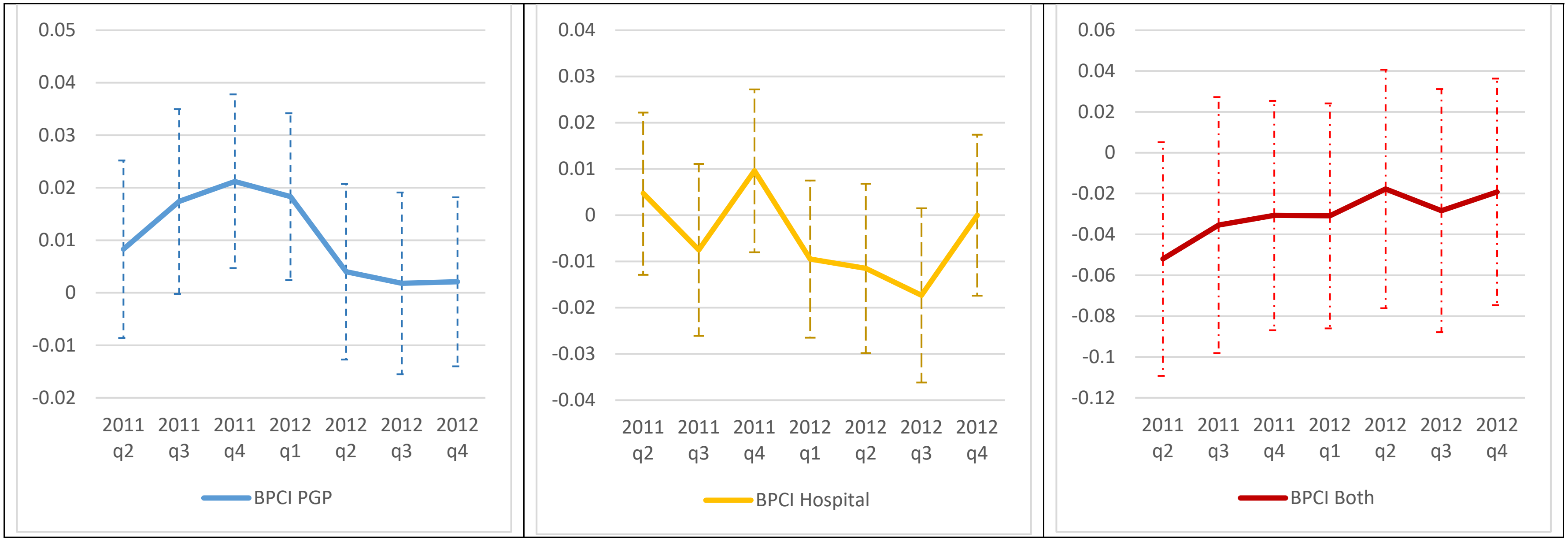

© 2022 Liao JM et al. *JAMA Health Forum.*

eFigure 2. Adjusted Parallel Trends for Surgical Episodes

Although the difference-in-differences assumption of parallel trends involves unobservable counterfactual values and is therefore untestable, we visually examined baseline period data for any suggestion of divergent trends in study outcomes between study groups prior to the start of the BPCI program. For primary and secondary outcomes, we also fit a series of generalized linear regression models in which dependent variables were outcomes and independent variables included time (quarter) fixed effects, a BPCI participant indicator variable (physician group or hospital), their interaction, as well as market (HRR) fixed effects, episode (DRG) fixed effects, and a vector of patient and market characteristics.

Total Episode Spending

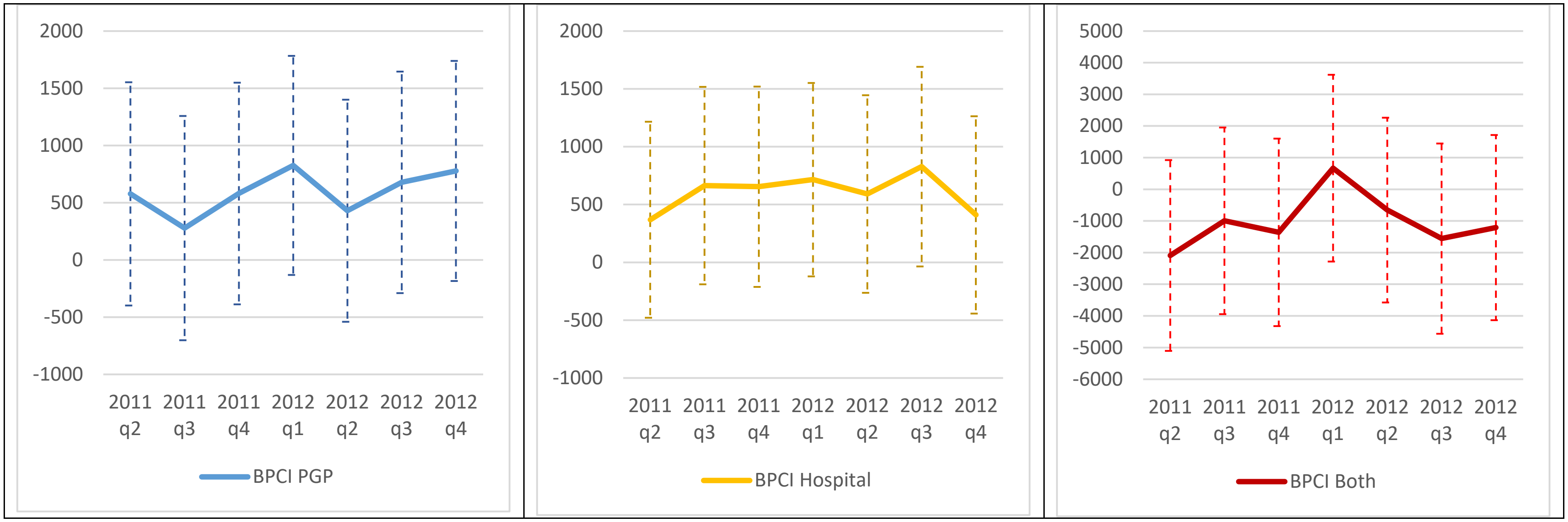

© 2022 Liao JM et al. *JAMA Health Forum.*

90-day Readmissions

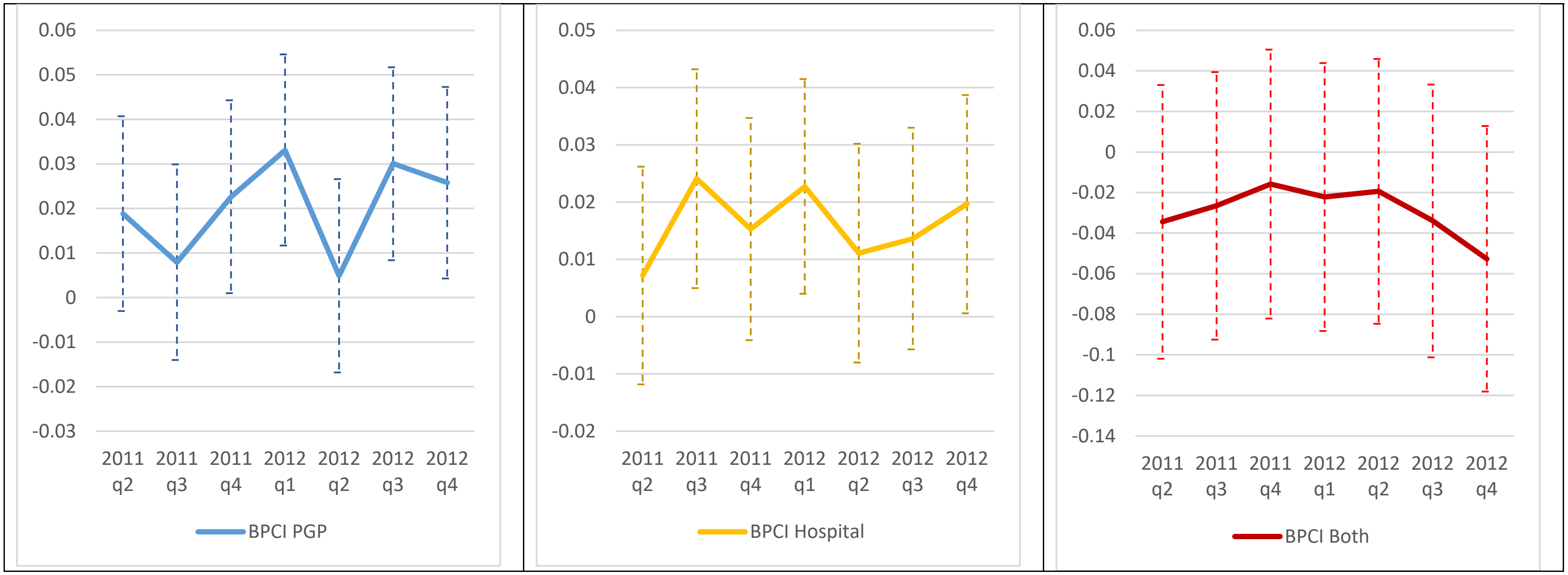

© 2022 Liao JM et al. *JAMA Health Forum.*

90-day Mortality

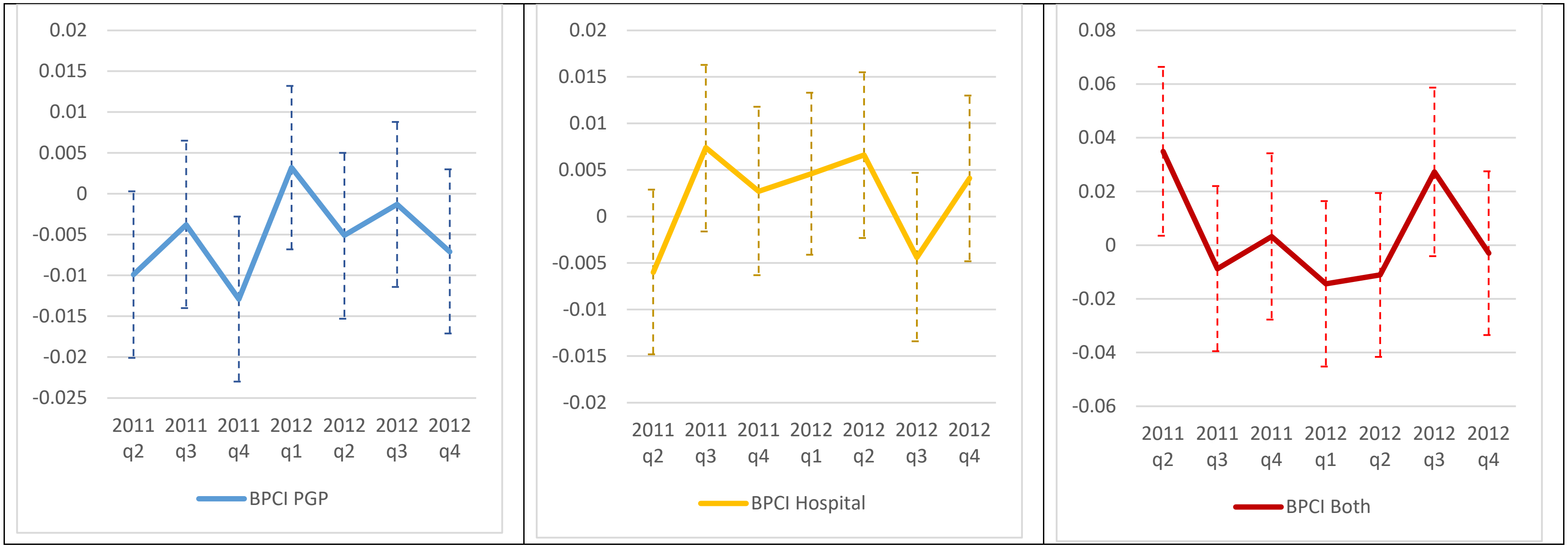

© 2022 Liao JM et al. *JAMA Health Forum.*

eMethods 2. Testing for Time-Varying Confounders in Difference-in-Difference Design

Following prior work[2-4], we examined whether the relationship between the primary outcomes and market-level covariates (Medicare Advantage penetration, Accountable Care Organization penetration, number of Medicare beneficiaries, hospital and skilled nursing facility concentration, hospital and skilled nursing facility bed counts) varied over time and whether the distributions of these variables changed differentially over time for BPCI versus non-BPCI physician groups and BPCI vs non-BPCI hospitals in propensity-matched samples.

We did so using models with the following specifications:

$$Y_{i,h(p),q,r} = X_{i,q,r} + X_{i,q,r} * T_q + T_q + HRR_r + DRG + Other\_Covar$$

For *i* patient, *h* hospital or *p* physician group, *q* quarter, *r* market

$X_{i,q,r}$ was each covariate of interest (market-level covariates), T_q was time (quarter) fixed effects, HRR_r was market fixed effects, *DRG* was MS-DRG fixed effects and *Other_Covar* was a vector of other patient and market characteristics. We evaluated the interaction between each covariate of interest and quarter fixed effects ($X_{i,q,r}*T_q$), for which corresponding coefficients provided evidence of time-varying relationships between the covariate and primary outcomes.

These analyses suggested time-varying relationships between primary outcomes and three market-level covariates – Medicare Advantage penetration, Accountable Care Organization penetration, and hospital concentration. Therefore, these terms ($MA_{pene}*T_q$, $ACO_{pene}*T_q$, and $Hospital_{conc}*T_q$) were considered time-varying confounders and included in final regression models (Appendix Methods 3).

© 2022 Liao JM et al. *JAMA Health Forum.*

eMethods 3. Model Specifications

We used a difference-in-differences method and a generalized linear models with identity links and normal distributions to estimate differential changes in outcomes for bundled payment versus non-bundled payment patients receiving care in organization-specific (physician group or hospital) baseline versus intervention periods.

Specifications:

$$Y_{i,h(p),q,r} = BPCI\_PGP\_ever_i + POST_{\frac{h}{p}} + BPCI\_Hosp\_ever_i + BPCI\_PGP\_ever_i * POST_{\frac{h}{p}} + BPCI_{Hosp_{ever}{}_i} * POST_{\frac{h}{p}} + BPCI_{Hosp_{ever}{}_i} * BPCI_{PGP_{ever}{}_i} + BPCI_{Hosp_{ever}{}_i} * BPCI_{PGP_{ever}{}_i} * POST_{\frac{h}{p}} + T_q + HRR_r + DRG + Covar + MA_{Pene} * T_q + ACO_{Pene} * T_q + Hospital_{conc} * T_q$$

For i patient, h hospital or p physician group, q quarter, r market

Our difference-in-differences estimators were interactions between dichotomous indicator variables reflecting BPCI participation by physician groups (*BPCI_PGP_ever*) and hospitals (*BPCI_Hosp_ever*) and the time period ($Post_{h/p}$):

- Patients receiving care through BPCI versus non-BPCI physician groups: $BPCI\_PGP\_ever*Post_{h/p}$
- Patients receiving care through BPCI versus non-BPCI hospitals: $BPCI\_Hosp\_ever*Post_{h/p}$
- Patients receiving care through both BPCI physician groups and hospitals versus neither BPCI physician groups or hospitals: $BPCI\_PGP\_ever*Post_{h/p} + BPCI\_Hosp\_ever*Post_{h/p} + BPCI\_PGP\_ever*BPCI\_Hosp\_ever*Post_{h/p}$

The interaction between BPCI physician group and hospital ($BPCI\_Hosp\_ever*BPCI\_Hosp\_ever*Post_{h/p}$) enabled us to compare the association between BPCI participation and outcomes for physician groups versus hospitals. T_q was time (quarter) fixed effects, HRR_r was market fixed effects, *DRG* was MS-DRG fixed effects. *Covar* was a vector of patient characteristics including age, sex, race/ethnicity, disability status, dual eligibility for Medicare and Medicaid, and 29 Elixhauser clinical conditions as well as market characteristics including Medicare Advantage penetration, Accountable Care Organization penetration, and number of Medicare beneficiaries. MA_{pene} was a market-level measure of Medicare Advantage penetration, ACO_{pene} was a market-level measure of Accountable Care Organization penetration, and $Hospital_{conc}$ was a market-level measure of hospital concentration – these three variables were interacted with T_q given confounder analysis (Appendix Methods 2).

© 2022 Liao JM et al. *JAMA Health Forum.*

eTable 1. Characteristics of BPCI and Non-BPCI Hospitals, Before and After Propensity Score Matching

	Before Matching			After Matching		
	Non-BPCI	BPCI	SMD	Non-BPCI	BPCI	SMD
**Hospitals, N**						
Overall	2248	385		1441	379	
By episode						
Congestive Heart Failure	2248	182		534	178	
Chronic Obstructive Pulmonary Disease, Bronchitis, or Asthma	2246	135		405	135	
Hip & Femur Procedures except Major Joint	2225	105		315	105	
Lower Extremity Joint Replacement	2239	311		918	306	
Percutaneous Coronary Intervention	1295	54		150	50	
Pneumonia & Pleurisy	2247	142		426	142	
Respiratory Infections & Inflammations	2247	75		225	75	
Sepsis	2245	125		372	124	
Spinal Fusion	1414	52		147	49	
Upper Extremity Joint Replacement	1869	39		114	38	
**HOSPITAL CHARACTERISTICS**						
**Ownership, N (%)**						
Not-for-profit	13287 (65.5)	913 (74.8)	0.4536	2700 (74.9)	902 (75.0)	0.0667
For-profit	4078 (20.1)	276 (22.6)		822 (22.8)	269 (22.4)	
Government	2910 (14.4)	31 (2.5)		84 (2.3)	31 (2.6)	
**Rural, N (%)**	5253 (25.9)	69 (5.7)	0.5783	167 (4.6)	69 (5.7)	-0.05
**Teaching Status, N (%)**						
Non-teaching	12563 (62.0)	580 (47.5)	0.2871	1771 (49.1)	579 (48.2)	0.0213
Minor teaching	5834 (28.8)	496 (40.7)		1456 (40.4)	487 (40.5)	
Major teaching	1878 (9.3)	144 (11.8)		379 (10.5)	136 (11.3)	
**Residents:beds ratio, Mean (SD)**	6.6 (19.8)	9.4 (21.0)	0.1336	8.2 (20.8)	8.9 (19.8)	0.0339

© 2022 Liao JM et al. *JAMA Health Forum.*

**Proportion of Medicare days*, Mean (SD)**	25.5 (8.8)	28.0 (7.2)	0.3126	28.1 (7.9)	27.9 (7.2)	-0.0164
**Total beds, Mean (SD)**	239.0 (199.5)	324.5 (232.8)	0.3944	301.8 (211.8)	315.4 (217.1)	0.0635
**Disproportionate share payment**, Mean (SD) $	3899263.3 (6007697.4)	5477123.5 (7237062.8)	0.2372	4899347.7 (6716490.7)	5269461.3 (6905124.6)	0.0543
**Health System Affiliated, N (%)**						
No	6931 (34.2)	210 (17.2)	0.3959	642 (17.8)	210 (17.5)	0.0087
Yes	13344 (65.8)	1010 (82.8)		2964 (82.2)	992 (82.5)	
**Bundled Payment Admissions**, Mean (SD)**	144.6 (159.1)	246.3 (210.8)	0.5444	227.4 (198.7)	240.7 (199.5)	0.0671
**Proportion of Discharges to highest volume SNF, Mean % (SD)**	35.1 (19.1)	27.4 (15.4)	-0.4438			-0.0006
**Proportion of Discharges to highest volume IRF, Mean % (SD)**	50.6 (45.3)	59.7 (42.0)	0.2088	59.7 (43.5)	59.6 (42.2)	-0.0022
**Hospital Market Share***, Mean (SD)**	10.4 (15.3)	14.4 (17.8)	0.2391	14.2 (18.2)	14.2 (17.5)	-0.0011
**MARKET CHARACTERISTICS**						
**Population, Mean (SD)**	2102826.5 (2050016.8)	2235764.4 (1908419.2)	0.0671	2174521.5 (2026878.7)	2231901.1 (1909378.2)	0.0291
**Admissions from low income areas****, Mean % (SD)**	23.0 (16.3)	19.0 (14.2)	-0.2628	18.8 (14.2)	19.0 (14.2)	0.0166
**MA penetration, Mean (SD)**	26.4 (13.2)	25.5 (11.8)	-0.0698	25.6 (13.1)	25.6 (11.8)	0.005
**SNF beds per 10000 patients, Mean (SD)**	10249.0 (8657.8)	10821.4 (9580.9)	0.0627	10600.7 (8828.5)	10801.6 (9610.5)	0.0218
**Hospital Concentration*****, Mean HHI (SD)**	1586.0 (1384.8)	1556.2 (1398.4)	-0.0214	1564.9 (1409.7)	1548.4 (1384.2)	-0.0118

Notes: BPCI=Bundled Payments for Care Improvement; HHI= Herfindahl-Hirschman Index; SMD=standardized mean difference. *Medicare days as a proportion of total inpatient days. **Number of BPCI admissions included in this analysis. ***A hospital's inpatient admission volume relative to total inpatient admission volume in a market. ****Defined as ZIP codes in which median household income <$40,000. *****HHI is a measure of market concentration that ranges from 0 to 10,000, with higher values corresponding to greater concentration.

© 2022 Liao JM et al. *JAMA Health Forum.*

eTable 2. Characteristics of Physicians in BPCI and Non-BPCI PGPs, Before and After Propensity Score Matching

	Before Matching			After Matching		
	Non-BPCI	BPCI	SMD	Non-BPCI	BPCI	SMD
**Physicians, N**						
Overall	71765	6689		24983	6423	
By episode						
Congestive Heart Failure	34631	2645		7512	2504	
Chronic Obstructive Pulmonary Disease, Bronchitis, or Asthma	32951	2990		8313	2771	
Hip & Femur Procedures except Major Joint	19338	1529		4194	1398	
Lower Extremity Joint Replacement	19298	1964		5091	1697	
Percutaneous Coronary Intervention	19135	741		2019	673	
Pneumonia & Pleurisy	35953	3246		8832	2944	
Respiratory Infections & Inflammations	25932	2012		5796	1932	
Sepsis	27179	2432		6639	2213	
Spinal Fusion	4546	188		519	173	
Upper Extremity Joint Replacement	3987	203		456	152	
**PHYSICIAN CHARACTERISTICS**						
**Sex, N (%)**						
Male	176448 (79.1)	13640 (76.0)	0.0757	36995 (74.9)	12363 (75.1)	-0.0044
Female	46502 (20.9)	4310 (24.0)		12376 (25.1)	4094 (24.9)	
**Age, Mean (SD)**	56.4 (10.3)	51.3 (8.4)	-0.5358	51.4 (9.0)	51.4 (8.5)	0.0057
**Annual services provided, Mean (SD)**	2338.6 (2205.9)	1596.8 (1280.5)	-0.4113	1572.4 (1484.3)	1582.0 (1294.8)	0.0069
**Payment for annual services, Mean (SD)**	218154.0 (206609.8)	162030.6 (124478.3)	-0.3291	158756.2 (152927.9)	159471.9 (124800.5)	0.0051
**Beneficiaries cared for, Mean (SD)**	563.5 (472.7)	481.7 (289.9)	-0.2088	469.0 (377.4)	476.0 (289.5)	0.0207

© 2022 Liao JM et al. *JAMA Health Forum.*

**Care for patients at a teaching hospital N (%)**						
No	85269 (38.2)	6171 (34.4)	0.0805	17033 (34.5)	5752 (35.0)	-0.0095
Yes	137681 (61.8)	11779 (65.6)		32338 (65.5)	10705 (65.0)	
**Rural, N (%)**	7047 (3.2)	356 (2.0)	0.0641	1004 (2.0)	347 (2.1)	0
**Affiliated with health system N (%)**						
No	17154 (7.7)	568 (3.2)	0.2009	1572 (3.2)	566 (3.4)	-0.0143
Yes	205796 (92.3)	17382 (96.8)		47799 (96.8)	15891 (96.6)	
**Proportion of services provided in the inpatient setting, Mean (SD) %**	51.7 (33.2)	77.5 (29.9)	0.8158	77.2 (29.3)	77.2 (29.8)	-0.0003
**Relative proportion of services provided in the outpatient setting*, Mean (SD) %**						
Lower	209567 (94.0)	17723 (98.7)	-0.2553	48756 (98.8)	16230 (98.6)	0.0117
Higher	13383 (6.0)	227 (1.3)		615 (1.2)	227 (1.4)	
**Proportion of admissions from dual-eligible individuals, Mean (SD) %**	31.0 (33.3)	33.4 (27.0)	0.0821	33.5 (31.1)	33.5 (27.1)	-0.0001
**Proportion of admissions from Black individuals, Mean (SD) %**	10.1 (22.5)	9.1 (17.5)	-0.0503	9.3 (19.4)	9.3 (17.9)	0.0032
**Proportion of BPCI-eligible admissions accounted for episodes of interest**, Mean (SD) %**	31.2 (27.1)	27.3 (23.9)	-0.1497	27.4 (24.7)	27.2 (23.6)	-0.0097
**Proportion of admissions accounted for by the highest volume hospital***, Mean (SD) %**	95.6 (12.7)	93.0 (15.9)	-0.1823	93.9 (14.7)	93.8 (14.9)	-0.0088
**TIN CHARACTERISTICS**						
**Physicians within a TIN, Mean (SD)**	16.8 (25.0)	32.6 (37.0)	0.5004	28.0 (30.7)	27.7 (31.0)	-0.0088
**Multi-specialty TIN, N (%)**	222314 (99.7)	17944 (100)	0.0632	49360 (100)	16451 (100)	-0.0083
**MARKET CHARACTERISTICS**						
**Population, Mean (SD)**	2237596.2 (2014241.0)	2392892.2 (1945824.8)	0.0784	2322459.2 (1989961.5)	2335682.1 (1907929.3)	0.0068
**Admissions from low income areas****, Mean % (SD)**	21.8 (15.8)	21.7 (15.3)	-0.0091	21.6 (15.6)	21.5 (15.1)	-0.0063
**MA penetration, Mean (SD)**	25.6 (12.6)	25.7 (11.6)	0.0075	25.9 (12.2)	25.8 (11.7)	-0.0076

© 2022 Liao JM et al. *JAMA Health Forum.*

**SNF beds per 10000 patients, Mean (SD)**	11150.7 (9025.5)	11061.4 (8774.7)	-0.01	10943.9 (8728.5)	10988.3 (8826.6)	0.0051
**Hospital Concentration*****, Mean HHI (SD)**	1522.0 (1384.7)	1478.1 (1378.4)	-0.0318	1498.3 (1423.7)	1493.2 (1386.4)	-0.0037

Notes: BPCI=Bundled Payments for Care Improvement; HHI= Herfindahl-Hirschman Index; SMD=standardized mean difference. *Physicians with greater than the median proportion of services provided in the outpatient setting defined as *higher*; physicians with proportions lower than the median define as *lower*. **Proportion of admissions among 48 BPCI-eligible episodes accounted for the by 10 episodes of interest in this analysis; averaged across 10 episodes of interest. ***Among all admissions by a physician to different hospitals, the proportion of admissions accounted for by the highest volume hospital. ****Defined as ZIP codes with median household income <$40,000. *****HHI is a measure of market concentration that ranges from 0 to 10,000, with higher values corresponding to greater concentration.

© 2022 Liao JM et al. *JAMA Health Forum.*

eFigure 3. Unadjusted Changes in Medical Episode Outcomes, 2011-2017

Panel A. Total Episode Spending

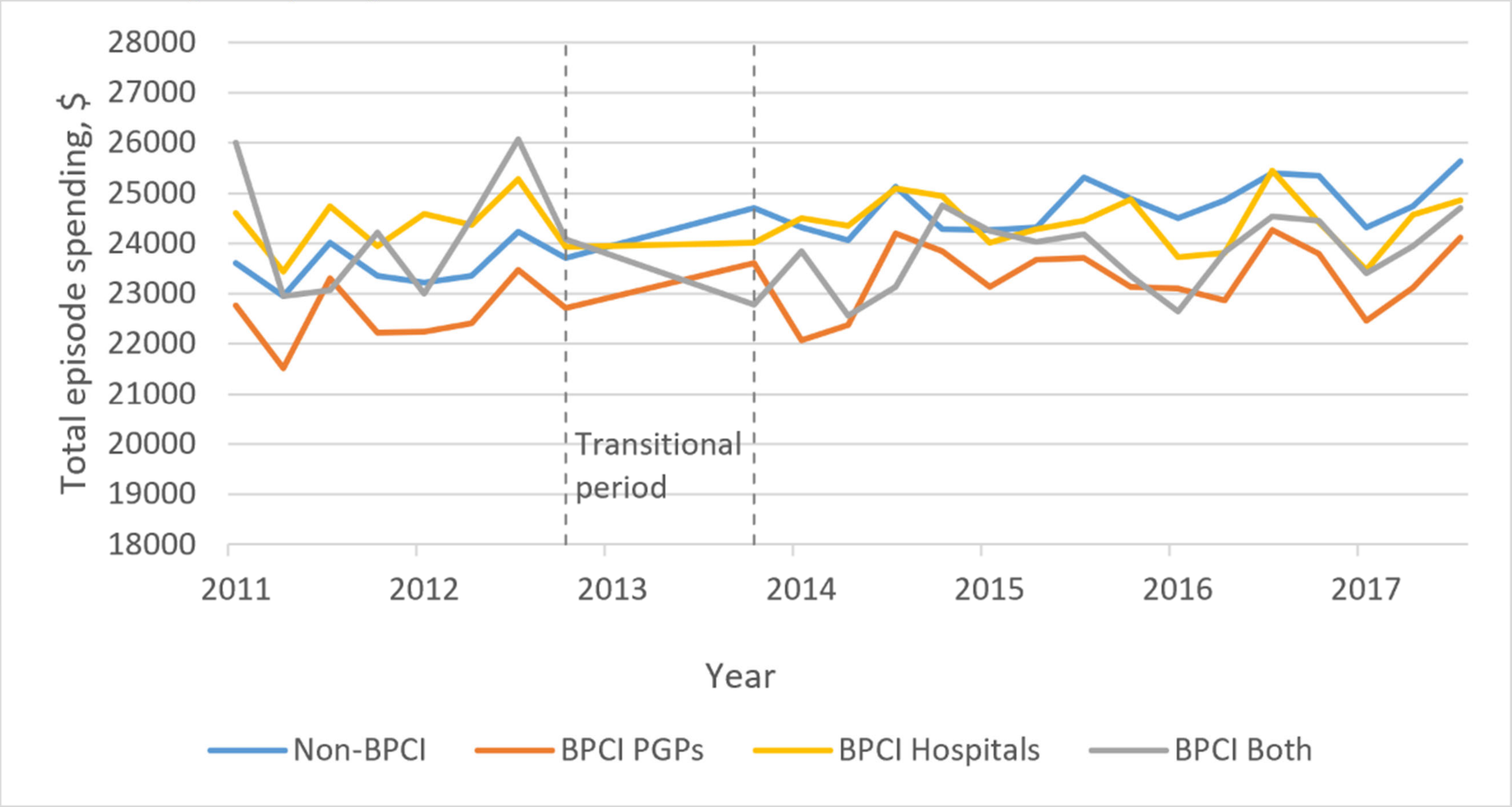

© 2022 Liao JM et al. *JAMA Health Forum.*

Panel B. 90-day Readmissions

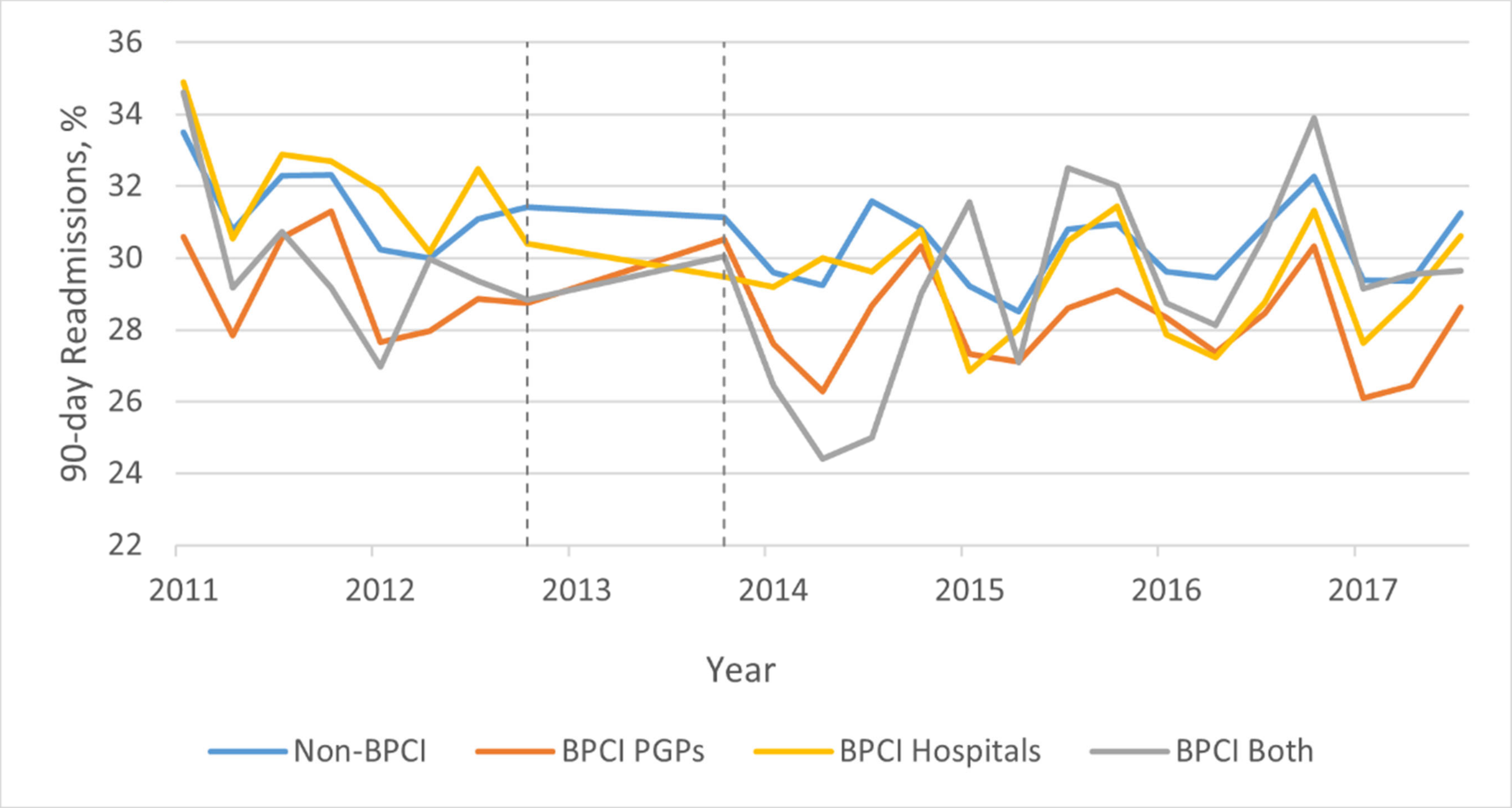

© 2022 Liao JM et al. *JAMA Health Forum.*

Panel C. 90-day Mortality

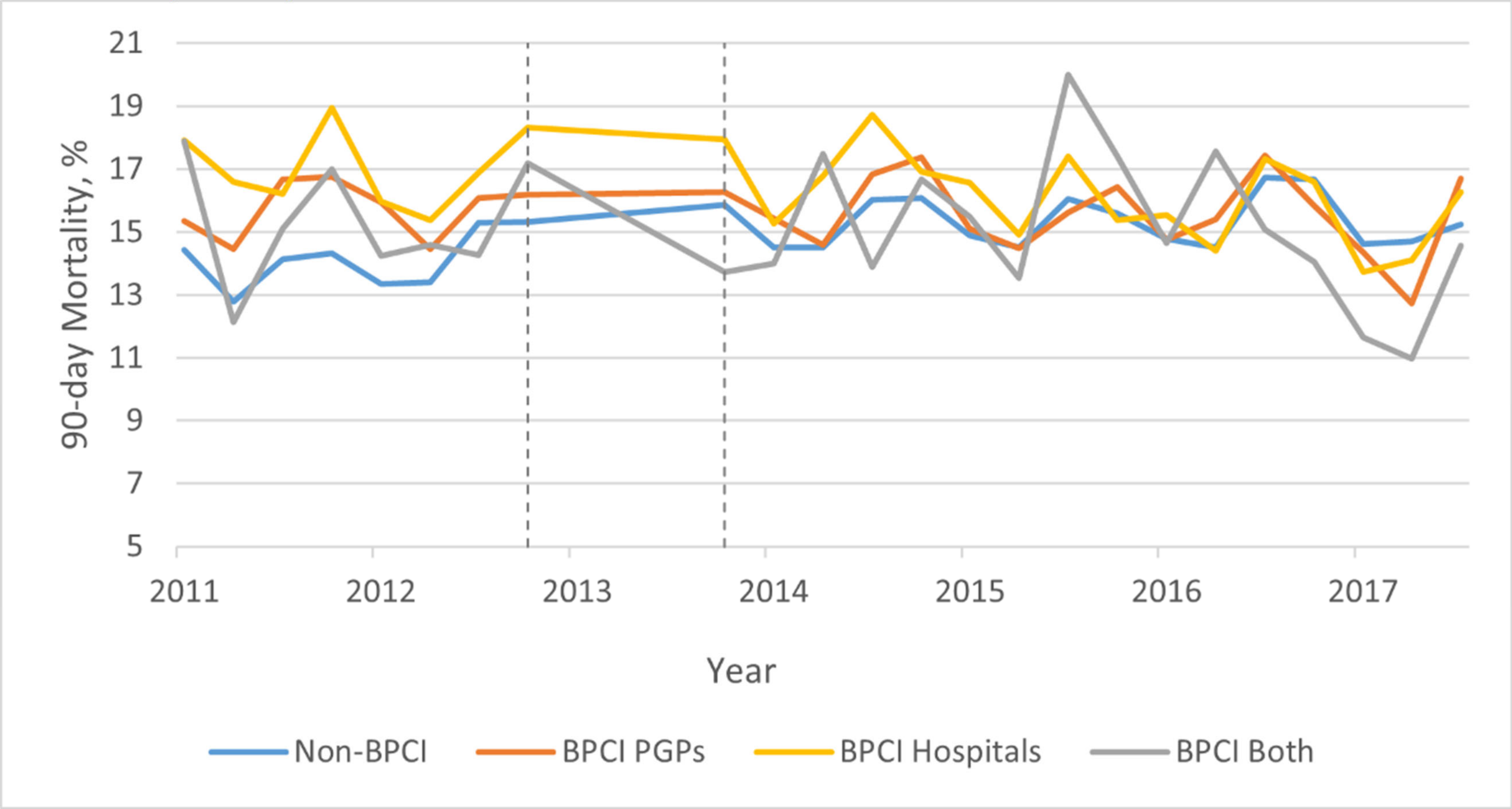

© 2022 Liao JM et al. *JAMA Health Forum.*

eTable 3. Characteristics of Medical Episode Patients and Markets, by Study Period and Bundled Payment Status

	Non-BPCI		BPCI PGP		BPCI Hospital		BPCI Both	
	**Pre**	**Post**	**Pre**	**Post**	**Pre**	**Post**	**Pre**	**Post**
**Beneficiaries, N**	299,246	180,332	44,470	21,199	34,765	25,265	3,983	2,552
**Episodes, N**	312,551	186,518	44,831	21,301	35,172	25,553	3,994	2,559
Congestive Heart Failure	67,322	47,514	8,387	4,728	10,057	8,067	968	639
Chronic Obstructive Pulmonary Disease, Bronchitis, or Asthma	64,925	33,534	9,967	4,635	7,093	4,713	756	500
Pneumonia & Pleurisy	92,256	45,173	14,336	6,009	10,749	6,296	1,321	740
Respiratory Infections & Inflammations	30,628	16,153	5,144	2,359	2,470	1,572	234	143
Sepsis	57,420	44,144	6,997	3,570	4,803	4,905	715	537
**PATIENT CHARACTERISTICS**								
**Sex, N (%)**								
Male	135785 (43.44)	82382 (44.17)	19579 (43.67)	9354 (43.91)	15243 (43.34)	11317 (44.29)	1788 (44.77)	1147 (44.82)
Female	176766 (56.56)	104136 (55.83)	25252 (56.33)	11947 (56.09)	19929 (56.66)	14236 (55.71)	2206 (55.23)	1412 (55.18)
**Age, Mean (SD)**	76.54 (12.07)	77.18 (11.86)	76.23 (12.12)	76.61 (11.86)	77.82 (11.98)	77.84 (11.81)	77.20 (12.30)	77.36 (11.68)
**Race, N (%)**								
White	262328 (83.93)	156279 (83.79)	37757 (84.22)	17907 (84.07)	28460 (80.92)	20490 (80.19)	3334 (83.48)	2106 (82.30)
Black	27702 (8.86)	16307 (8.74)	3653 (8.15)	1739 (8.16)	3518 (10.00)	2503 (9.80)	326 (8.16)	208 (8.13)
Other*	22521 (7.21)	13932 (7.47)	3421 (7.63)	1655 (7.77)	3194 (9.08)	2560 (10.02)	334 (8.36)	245 (9.57)
**Disabled, N (%)**	42910 (13.73)	23261 (12.47)	6403 (14.28)	2833 (13.30)	4107 (11.68)	2933 (11.48)	533 (13.35)	303 (11.84)
**Medicare/Medicaid Dual Eligible, N (%)**	84864 (27.15)	46818 (25.10)	12633 (28.18)	5544 (26.03)	9368 (26.63)	6405 (25.07)	1029 (25.76)	618 (24.15)

© 2022 Liao JM et al. *JAMA Health Forum.*

**Elixhauser Comorbidity Index, mean (SD) points**	20.09 (14.05)	20.24 (14.03)	19.46 (14.03)	18.91 (13.97)	20.50 (13.97)	20.03 (14.00)	20.35 (14.03)	19.48 (13.96)
**Prior IRF use, mean (SD) %[**]**	3.36 (18.01)	3.88 (19.31)	3.19 (17.56)	3.72 (18.92)	3.62 (18.67)	3.64 (18.73)	2.90 (16.79)	2.81 (16.54)
**Prior SNF use, mean (SD) %[**]**	19.55 (39.66)	20.10 (40.08)	17.06 (37.62)	16.60 (37.21)	19.79 (39.85)	19.07 (39.29)	18.48 (38.82)	18.60 (38.92)
**Prior hospital use, mean (SD) %[**]**	52.89 (49.92)	56.99 (49.51)	47.63 (49.94)	51.61 (49.98)	49.68 (50.00)	52.47 (49.94)	45.89 (49.84)	46.97 (49.92)
**MARKET CHARACTERISTICS**								
**Number of Medicare FFS beneficiaries, Mean (SD)**	158032.0 (116628.2)	162580.6 (119958.0)	148851.5 (113259.7)	158133.5 (121180.8)	160254.4 (126964.7)	157037.3 (122859.1)	184535.9 (151476.9)	204221.0 (165038.8)
**Quarterly Admissions for BPCI-eligible episodes[***], Mean (SD)**	550.49 (465.94)	565.88 (511.05)	518.76 (451.20)	525.95 (476.59)	574.25 (515.68)	554.98 (517.65)	669.74 (629.07)	739.37 (690.81)
**ACO penetration, Mean (SD) %**	7.24 (11.65)	24.09 (13.41)	7.16 (11.47)	23.91 (13.06)	7.05 (12.07)	25.97 (13.53)	7.41 (12.05)	25.56 (12.80)
**MA penetration, Mean (SD) %**	27.22 (12.32)	32.28 (12.28)	26.23 (11.59)	32.02 (11.64)	26.85 (11.64)	32.12 (11.45)	24.99 (10.87)	28.79 (11.24)
**Hospital Concentration[****], Mean (SD) HHI**	1182.50 (967.12)	1195.31 (957.85)	1253.95 (965.28)	1269.27 (974.04)	1211.68 (917.40)	1203.87 (891.94)	1166.88 (854.88)	1147.46 (939.90)
**SNF Concentration[****], Mean (SD) HHI**	280.45 (237.82)	279.13 (240.06)	300.62 (259.21)	299.81 (268.67)	288.71 (225.27)	289.26 (226.17)	284.98 (217.16)	274.42 (225.02)
**Hospital Supply[*****], Mean (SD) beds**	6856.02 (6313.62)	6960.35 (6421.79)	6303.64 (5554.41)	6764.02 (6078.47)	6960.30 (6800.47)	6762.47 (6542.29)	7178.30 (6256.37)	7841.91 (6755.81)
**SNF Supply[*****], Mean (SD) beds**	12696.14 (10734.30)	12918.58 (10971.21)	12390.06 (11039.55)	13382.01 (12028.64)	12986.53 (12315.07)	12754.32 (11809.15)	15299.40 (15057.82)	17166.56 (16278.27)

Notes: ACO=Accountable Care Organization; BPCI=Bundled Payments for Care Improvement; FFS=fee-for-service; HHI= Herfindahl-Hirschman Index; IRF=Inpatient Rehabilitation Facility; MA=Medicare Advantage; PGP=Physician Group Practice; SMD=standardized mean difference; SNF=Skilled Nursing Facility. [*]Includes Asian, North American Native, and other categories as reported in the Medicare claims data. [**]Use within the last 12 months.[***]Number of admissions for the 48 episodes included in the BPCI program. [****]HHI is a measure of market concentration that ranges from 0 to 10,000, with higher values corresponding to greater concentration. [*****]Defined by total number of beds per 10,000 Medicare beneficiaries in a market.

© 2022 Liao JM et al. *JAMA Health Forum.*

eTable 4. Characteristics of Surgical Episode Patients and Markets, by Study Period and Bundled Payment Status

	Non-BPCI		BPCI PGP		BPCI Hospital		BPCI Both	
	Pre	Post	Pre	Post	Pre	Post	Pre	Post
**Beneficiaries, N**	205,986	153,398	27,624	21,078	35,446	32,746	2,970	3,585
**Episodes, N**	207,716	154,431	27,658	21,096	35,480	32,780	2,971	3,586
Hip & Femur Procedures except Major Joint	24,295	15,272	3,687	1,576	3,412	2,496	219	119
Lower Extremity Joint Replacement	147,774	117,156	19,619	16,613	27,782	27,176	2,662	3,379
Percutaneous Coronary Intervention	17,924	9,005	1,654	745	2,489	1,483	38	19
Spinal Fusion	11,737	7,852	1,574	1,184	1,392	1,058	43	47
Upper Extremity Joint Replacement	5,986	5,146	1,124	978	405	567	9	22
**PATIENT CHARACTERISTICS**								
**Sex, N (%)**								
Male	76621 (36.89)	57651 (37.33)	10104 (36.53)	7970 (37.78)	12635 (35.61)	11790 (35.97)	1020 (34.33)	1319 (36.78)
Female	131081 (63.11)	96785 (62.67)	17555 (63.47)	13125 (62.22)	22846 (64.39)	20988 (64.03)	1951 (65.67)	131081 (63.11)
**Age, Mean (SD)**	74.92 (9.57)	74.64 (9.24)	74.68 (9.04)	73.67 (8.24)	75.05 (9.50)	74.48 (9.04)	74.92 (8.73)	74.92 (9.57)
**Race, N (%)**								
White	184823 (88.98)	137056 (88.75)	25057 (90.60)	18919 (89.68)	30890 (87.06)	28223 (86.10)	2640 (88.86)	3187 (88.87)
Black	11166 (5.38)	8047 (5.21)	1293 (4.67)	1047 (4.96)	2170 (6.12)	1996 (6.09)	196 (6.60)	218 (6.08)

© 2022 Liao JM et al. *JAMA Health Forum.*

Other*	11727 (5.65)	9328 (6.04)	1308 (4.73)	1130 (5.36)	2420 (6.82)	2561 (7.81)	135 (4.54)	181 (5.05)
**Disabled, N (%)**	18987 (9.14)	13066 (8.46)	2051 (7.42)	1390 (6.59)	2924 (8.24)	2513 (7.67)	194 (6.53)	242 (6.75)
**Medicare/Medicaid Dual Eligible, N (%)**	27923 (13.44)	18074 (11.70)	3041 (11.00)	1720 (8.15)	4643 (13.09)	3760 (11.47)	298 (10.03)	269 (7.50)
**Elixhauser Comorbidity Index, Mean (SD) points**	7.23 (11.85)	6.27 (11.86)	5.78 (11.06)	4.10 (10.36)	6.47 (11.36)	5.31 (11.32)	5.87 (11.21)	3.51 (9.87)
**Prior IRF use, mean (SD) %****	2.15 (14.50)	1.97 (13.88)	1.36 (11.60)	0.91 (9.47)	1.90 (13.65)	1.50 (12.16)	1.51 (12.22)	0.86 (9.26)
**Prior SNF use, mean (SD) %****	7.73 (26.70)	7.23 (25.89)	6.06 (23.87)	4.22 (20.11)	7.20 (25.84)	5.99 (23.72)	7.07 (25.63)	4.13 (19.89)
**Prior hospital use, mean (SD) %****	29.75 (45.72)	32.91 (46.99)	27.50 (44.65)	30.92 (46.22)	25.43 (43.55)	26.33 (44.04)	24.17 (42.82)	23.84 (42.62)
**MARKET CHARACTERISTICS**								
**Number of Medicare FFS beneficiaries, Mean (SD)**	152790.6 (115850.4)	156964.4 (118205.1)	142610.3 (106822.9)	153508.9 (113755.7)	152703.8 (109355.5)	160574.7 (112089.1)	184233.3 (121056.8)	188914.1 (117773.7)
**Quarterly Admissions for BPCI-eligible episodes***, Mean (SD)**	477.36 (430.41)	564.42 (504.63)	445.23 (407.03)	573.35 (507.18)	491.73 (400.04)	593.91 (476.34)	675.15 (485.21)	810.52 (535.56)
**ACO penetration, Mean (SD) %**	6.04 (10.46)	22.73 (13.35)	6.33 (11.07)	24.00 (13.31)	7.63 (12.75)	26.36 (13.55)	6.53 (11.24)	26.91 (13.01)
**MA penetration, Mean (SD) %**	25.92 (12.06)	31.11 (12.26)	27.30 (11.63)	32.59 (11.55)	26.80 (12.58)	31.87 (12.06)	25.69 (11.51)	30.23 (10.68)
**Hospital Concentration****, Mean (SD) HHI**	1243.07 (983.26)	1242.43 (952.23)	1421.06 (1181.41)	1381.81 (1133.07)	1192.44 (948.91)	1163.00 (890.56)	906.68 (627.86)	907.52 (579.00)

© 2022 Liao JM et al. *JAMA Health Forum.*

**SNF Concentration****, Mean (SD) HHI**	300.09 (263.59)	293.67 (263.33)	322.13 (259.41)	300.25 (239.75)	285.69 (218.33)	278.60 (216.41)	223.24 (161.04)	210.10 (168.91)
**Hospital Supply*****, Mean (SD) beds**	6588.50 (6178.49)	6691.94 (6224.16)	5836.27 (5442.13)	6287.84 (5739.90)	6809.93 (6435.15)	7076.01 (6516.75)	7807.79 (6027.81)	7788.32 (5732.02)
**SNF Supply*****, Mean (SD) beds**	12181.33 (10732.68)	12457.89 (10941.69)	11273.84 (10193.95)	12339.01 (10847.54)	12167.34 (10681.18)	12767.57 (10751.25)	16021.88 (13314.63)	16653.10 (12624.25)

Notes: ACO=Accountable Care Organization; BPCI=Bundled Payments for Care Improvement; FFS=fee-for-service; HHI= Herfindahl-Hirschman Index; IRF=Inpatient Rehabilitation Facility; MA=Medicare Advantage; PGP=Physician Group Practice; SMD=standardized mean difference; SNF=Skilled Nursing Facility. *Includes Asian, North American Native, and other categories as reported in the Medicare claims data. **Use within the last 12 months.***Number of admissions for the 48 episodes included in the BPCI program. ****HHI is a measure of market concentration that ranges from 0 to 10,000, with higher values corresponding to greater concentration. *****Defined by total number of beds per 10,000 Medicare beneficiaries in a market.

© 2022 Liao JM et al. *JAMA Health Forum.*

eTable 5. Unadjusted Changes in Medical Episode Outcomes, Baseline versus Intervention Period

	**Non-BPCI**		**BPCI PGP**		**BPCI Hospital**		**BPCI Both**	
	**Pre**	**Post**	**Pre**	**Post**	**Pre**	**Post**	**Pre**	**Post**
**Total episode spending, Mean $**	23,941	24,861	22,903	23,210	24,389	24,335	23,952	23,913
**90-day Readmissions, Mean %**	30.9	30.4	28.9	27.9	30.9	29.3	29.0	30.1
**90-day Mortality, Mean %**	14.6	15.3	15.8	15.1	16.9	15.7	15.4	14.7
**Exploratory Outcomes**								
Spending, $								
Institutional PAC*	5,721	5,833	5,522	5,316	6,397	5,598	6,130	5,602
HHA	1,265	1,371	1,239	1,334	1,292	1,456	1,346	1,396
Utilization								
Discharge to institutional PAC Providers**, Mean %	20.2	21.1	19.6	19.0	22.5	21.8	23.0	23.0
Discharge with HHA, Mean %	7.9	7.0	7.5	7.2	8.3	8.0	9.5	8.4
SNF LOS, Mean days	9.4	9.3	9.2	8.4	10.3	9.0	10.1	9.0
HHA days, Mean days	6.0	6.2	6.0	6.2	6.2	6.7	6.5	6.2

Notes: BPCI=Bundled Payments for Care Improvement; ED=Emergency Department; HHA=Home Health Agency; LOS=Length of Stay; LTAC=Long-Term Acute Care; PAC=Post-Acute Care; PGP=Physician Group Practice; SNF=Skilled Nursing Facility. *Combination of SNF, inpatient rehabilitation facility, and long-term acute care spending. **Discharge to skilled nursing facility or inpatient rehabilitation facility.

© 2022 Liao JM et al. *JAMA Health Forum.*

eTable 6. Percent Changes in Medical Episode Outcomes, Baseline versus Intervention Period

	Non-BPCI	BPCI PGP	BPCI Hospital	BPCI Both	BPCI PGP vs Non-BPCI difference	BPCI Hospital vs Non-BPCI difference	BPCI Both vs Non-BPCI difference
	% change				$ or percentage point change		
**Total episode spending**	0.7	0.4	-1.9	0.3	-101	-763	-126
**90-day Readmissions**	0.6	0.4	-1.8	7.6	-0.1	-0.8	2.3
**90-day Mortality**	2.3	-1.6	-4.0	1.8	-0.7	-1.1	-0.1
**Exploratory Outcomes**							
Spending							
Institutional PAC*	1.2	-0.5	-8.1	-2.4	-141	-779	-294
HHA	1.1	1.1	9.6	-0.9	-0.17	92	-22
Utilization							
Discharge to institutional PAC providers**	-0.4	-0.7	0.0	-2.6	-0.2	0.2	-1.3
Discharge with HHA	-0.6	0.6	6.6	-3.0	0.4	2.1	-0.7
SNF LOS	1.0	-0.8	-8.0	-4.4	-0.2	-1.0	-0.6
HHA days	1.7	2.4	8.4	-2.1	0.04	0.4	-0.3

Notes: BPCI=Bundled Payments for Care Improvement; ED=Emergency Department; HHA=Home Health Agency; LOS=Length of Stay; PAC=Post-Acute Care; PGP=Physician Group Practice; SNF=Skilled Nursing Facility. *Combination of SNF, inpatient rehabilitation facility, and long-term acute care spending. **Discharge to skilled nursing facility or inpatient rehabilitation facility.

© 2022 Liao JM et al. *JAMA Health Forum.*

eFigure 4. Adjusted Changes in Medical Episode Outcomes Associated with Bundled Payment Participation Among PGPs and Hospitals

Panel A. Total Episode Spending

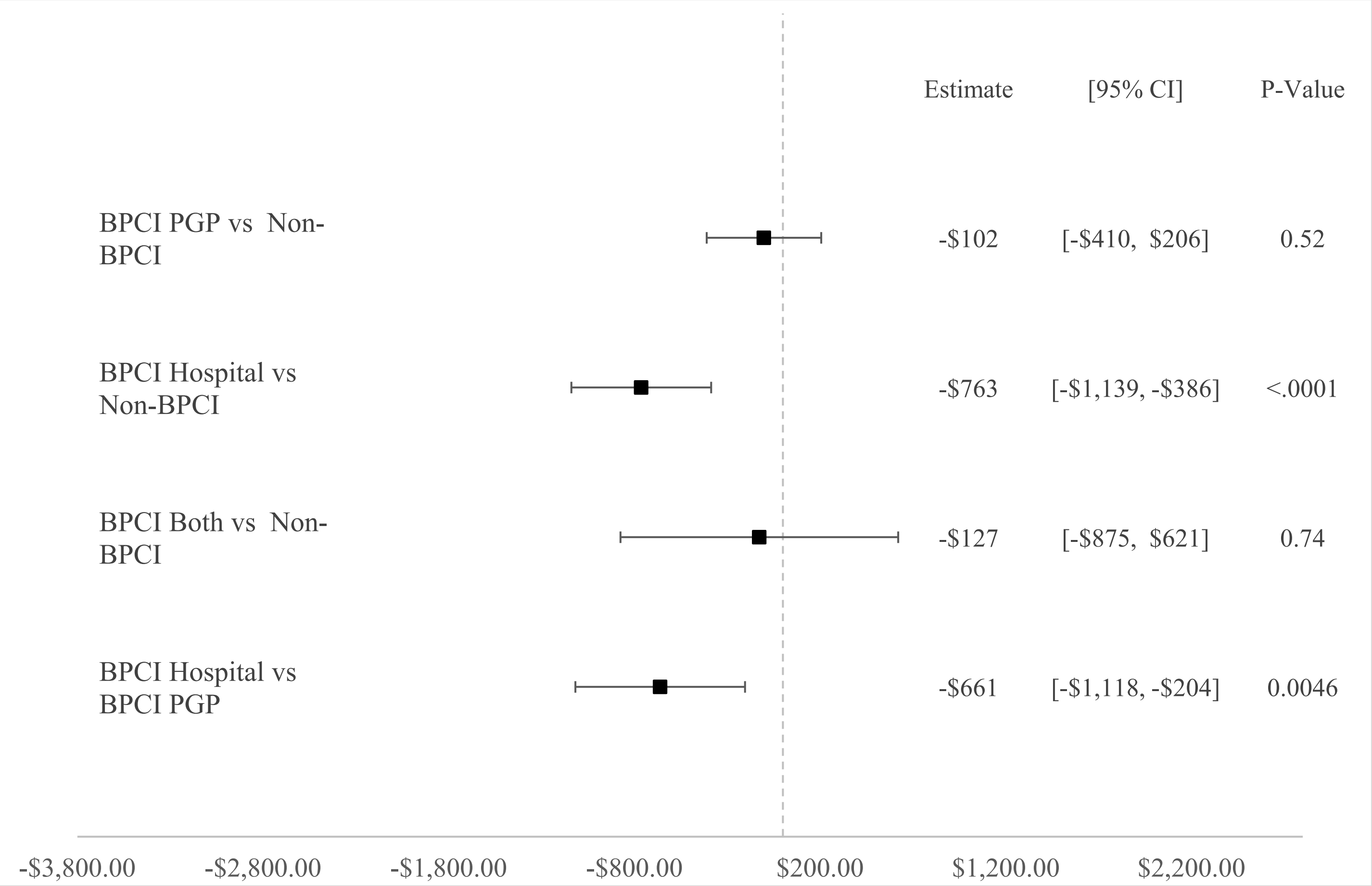

Notes: BPCI=Bundled Payments for Care Improvement; PGP=Physician Group Practice. Holm-Bonferroni correction was applied to the primary study outcome with corrected alpha as follows: BPCI Hospital vs Non-BPCI (p=0.0125; reject the null hypothesis), BPCI PGP vs Non-BPCI (p=0.025; do not reject the null hypothesis), BPCI Both vs Non-BPCI (p=0.05; do not reject the null hypothesis), BPCI Hospital vs BPCI PGP (0.017; reject the null hypothesis).

© 2022 Liao JM et al. *JAMA Health Forum.*

Panel B. 90-day Readmissions and Mortality

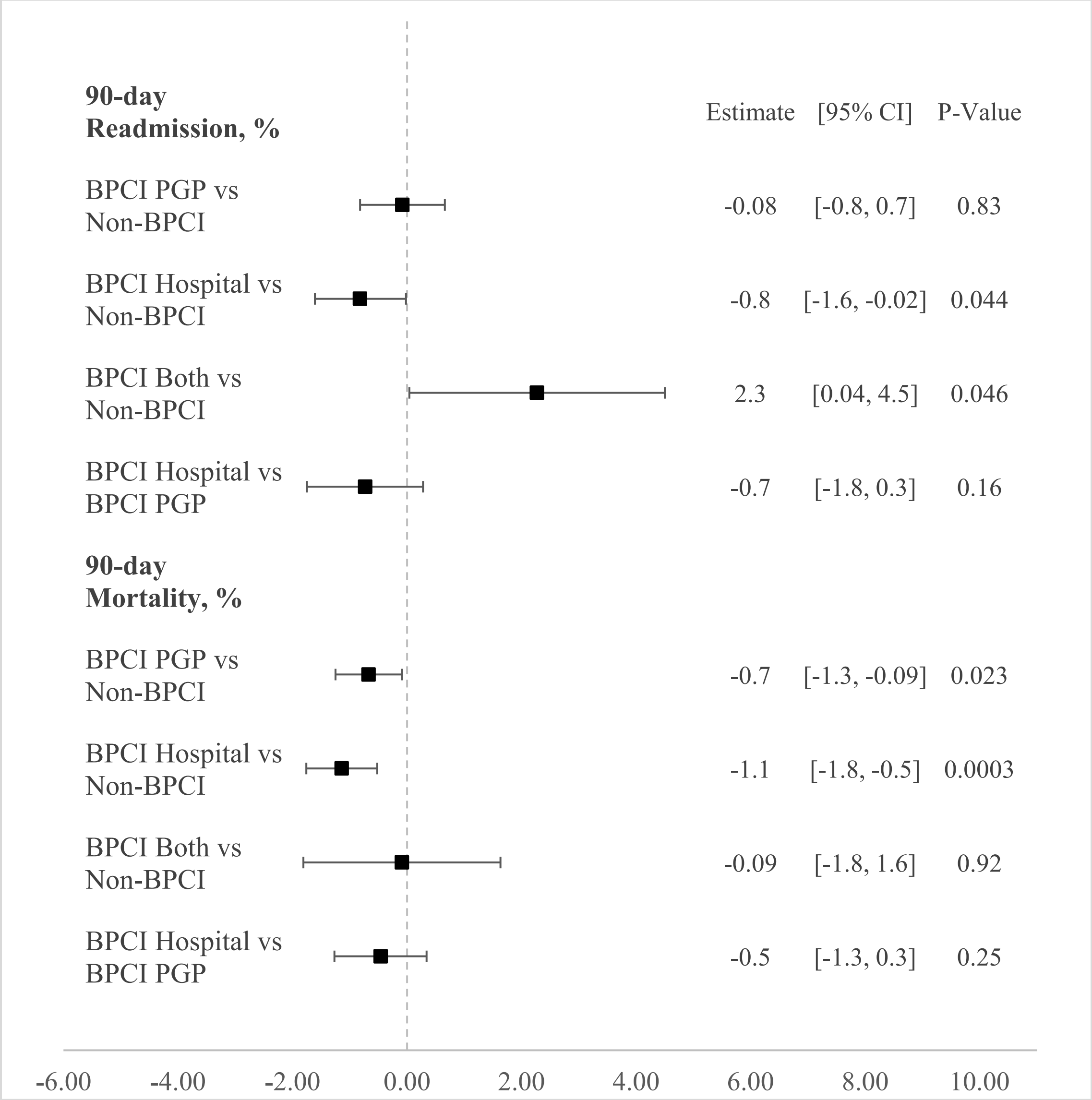

Notes: BPCI=Bundled Payments for Care Improvement; PGP=Physician Group Practice.

© 2022 Liao JM et al. *JAMA Health Forum.*

eTable 7. Adjusted Changes in Medical Episode Exploratory Outcomes Associated With Bundled Payments

	BPCI PGP vs Non-BPCI	BPCI Hospital vs Non-BPCI	BPCI Both vs Non-BPCI	BPCI Hospital vs BPCI PGP
**Spending, $**				
Institutional PAC*	-141	-779***	-294	-638***
HHA	$0	92	-22	92***
**Utilization, percentage point**				
Discharge to Institutional PAC Providers**	-0.6	-1.1***	-0.1	-0.5
Discharge with HHA	0.4	2.1***	-0.7	1.7***
SNF LOS	-0.2	-1.0***	-0.6	-0.8***
HHA days	0.04	0.4***	-0.3	0.4***

Notes: BPCI=Bundled Payments for Care Improvement; ED=Emergency Department; HHA=Home Health Agency; LOS=Length of Stay; LTAC=Long-Term Acute Care; PAC=Post-Acute Care; PGP=Physician Group Practice; SNF=Skilled Nursing Facility. *Combination of SNF, inpatient rehabilitation facility, and long-term acute care spending. **Discharge to skilled nursing facility or inpatient rehabilitation facility. ***p<0.05.

© 2022 Liao JM et al. *JAMA Health Forum.*

eFigure 5. Unadjusted Changes in Surgical Episode Outcomes, 2011-2017

Panel A. Total Episode Spending

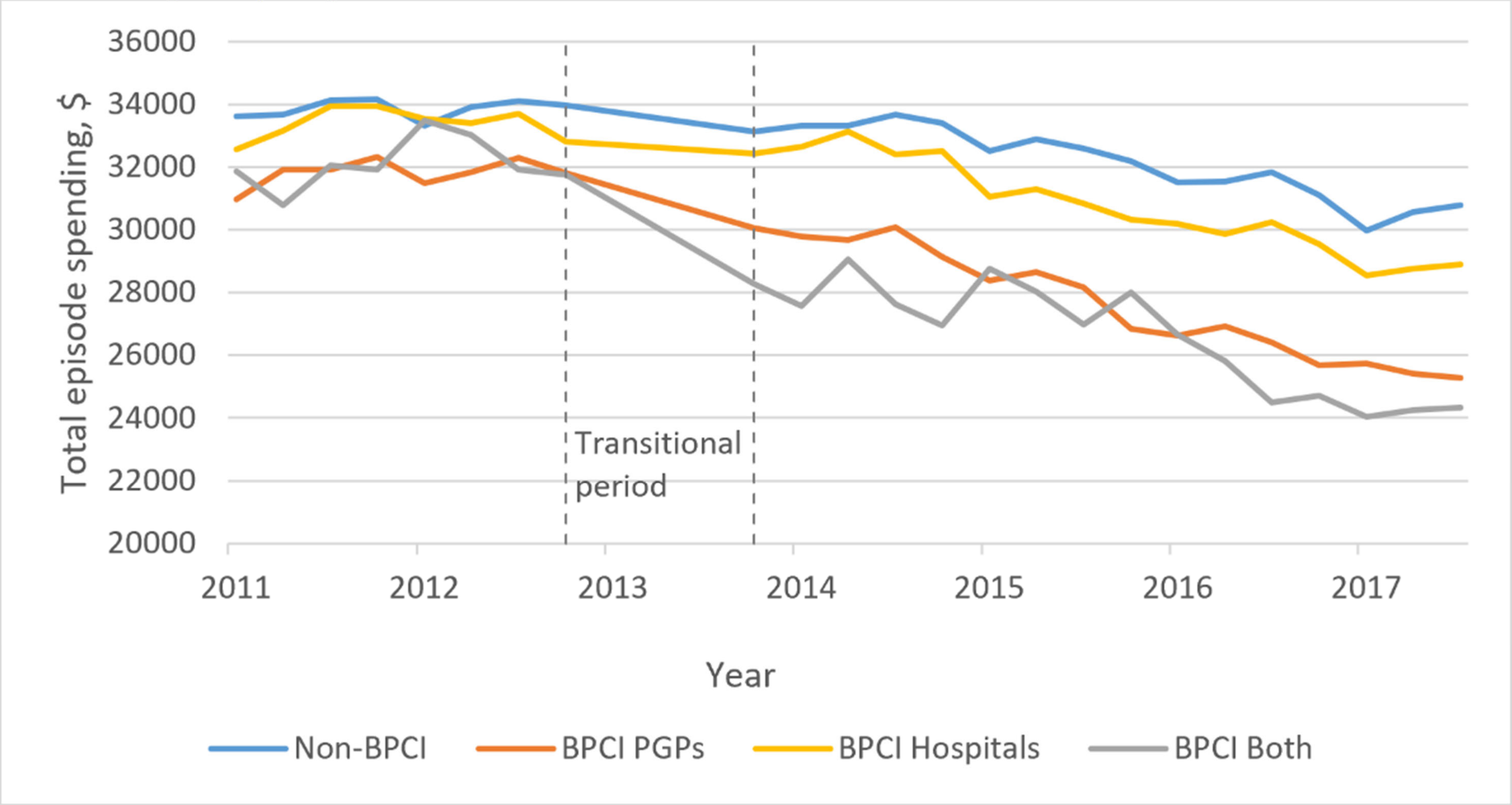

© 2022 Liao JM et al. *JAMA Health Forum.*

Panel B. 90-day Readmissions

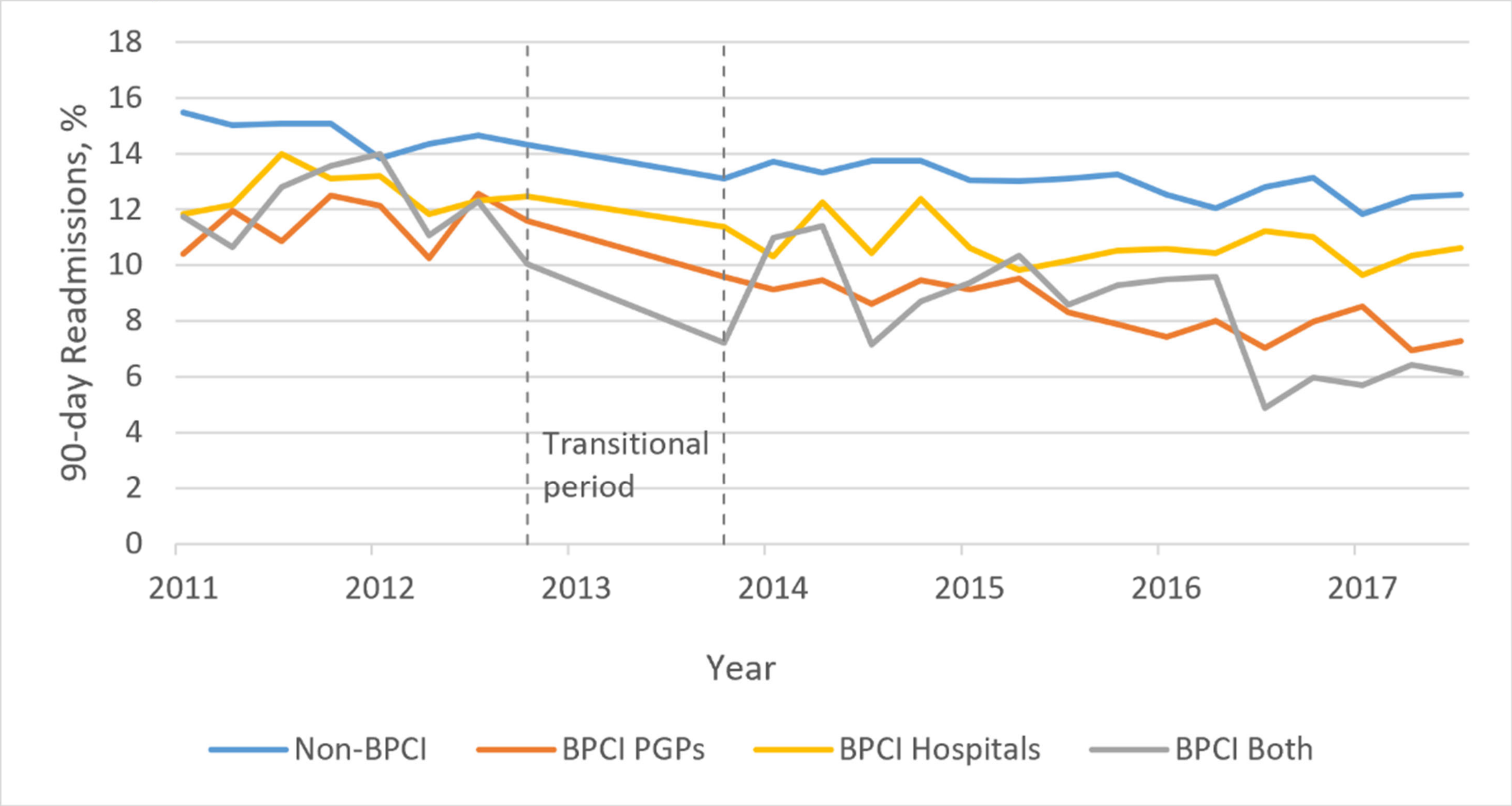

© 2022 Liao JM et al. *JAMA Health Forum.*

Panel C. 90-day Mortality

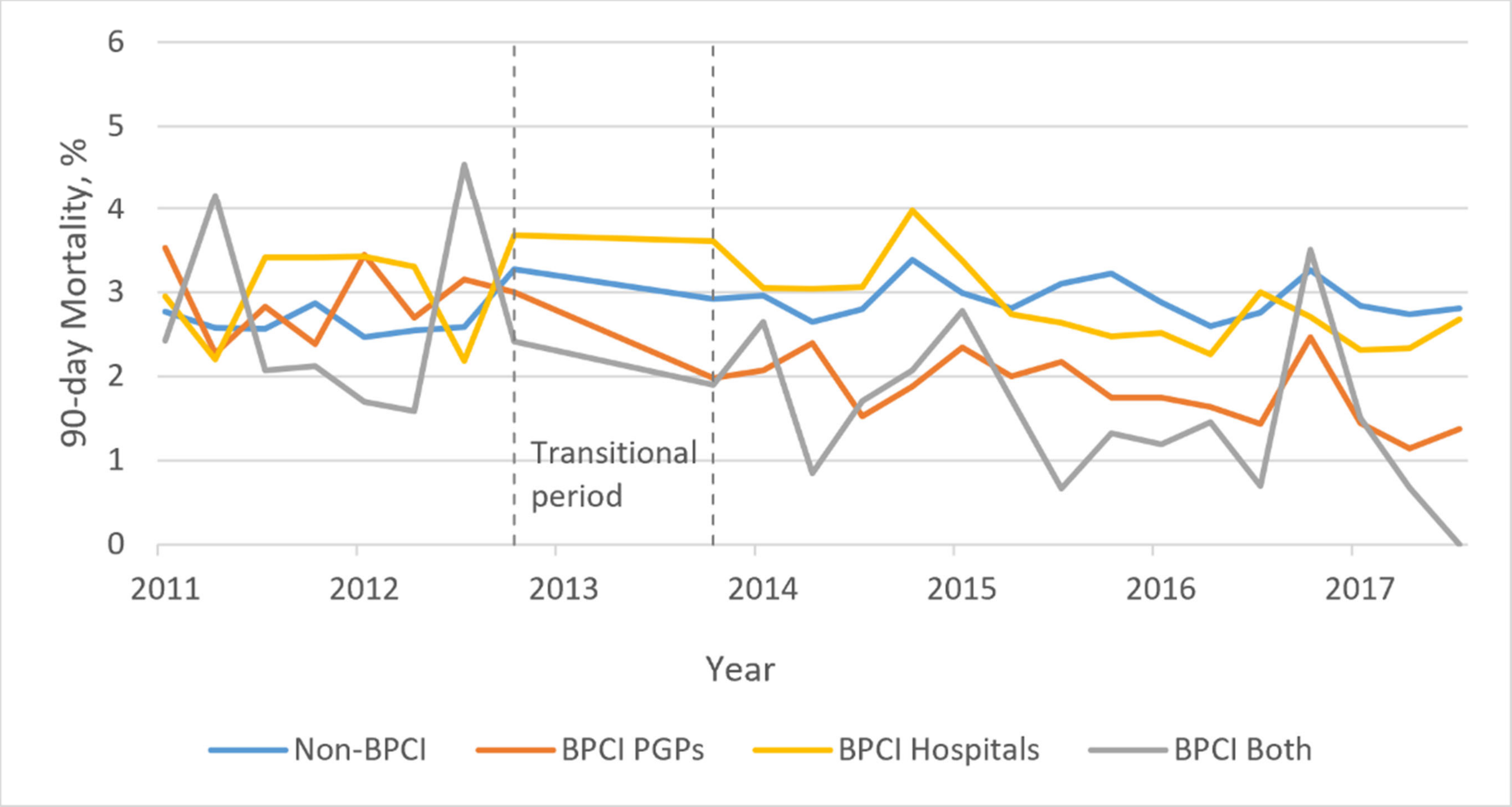

© 2022 Liao JM et al. *JAMA Health Forum.*

eTable 8. Unadjusted Changes in Surgical Episode Outcomes, Baseline versus Intervention Period

	Non-BPCI		BPCI PGP		BPCI Hospital		BPCI Both	
	**Pre**	**Post**	**Pre**	**Post**	**Pre**	**Post**	**Pre**	**Post**
**Total episode spending, Mean $**	33,557	31,629	30,987	26,515	33,135	29,870	31,055	25,735
**90-day Readmissions, Mean %**	14.2	12.7	10.7	7.8	12.1	10.5	11.2	7.7
**90-day Mortality, Mean %**	2.8	2.9	2.6	1.6	3.3	2.5	2.5	1.3
**Exploratory Outcomes**								
Spending								
Institutional PAC*	9,397	7,623	7,826	4,128	9,592	6,473	8,102	3,810
HHA	2,031	2,078	1,885	1,618	2,184	2,275	2,511	2,041
Utilization								
Discharge to institutional PAC Providers**, Mean %	46.9	39.6	41.9	26.1	49.8	37.1	46.8	27.0
Discharge with HHA, Mean %	18.3	22.0	20.6	23.7	20.6	27.4	28.0	32.9
SNF LOS, Mean days	13.4	11.2	11.9	6.5	13.5	9.6	11.1	6.1
HHA days, Mean days	8.1	7.7	7.2	5.5	8.6	8.3	9.6	7.0

Notes: BPCI=Bundled Payments for Care Improvement; ED=Emergency Department; HHA=Home Health Agency; LOS=Length of Stay; LTAC=Long-Term Acute Care; PAC=Post-Acute Care; SNF=Skilled Nursing Facility. *Combination of SNF, inpatient rehabilitation facility, and long-term acute care spending. **Discharge to skilled nursing facility or inpatient rehabilitation facility.

© 2022 Liao JM et al. *JAMA Health Forum.*

eTable 9. Percent Changes in Surgical Episode Outcomes, Baseline versus Intervention Period

	Non-BPCI	BPCI PGP	BPCI Hospital	BPCI Both	BPCI PGP vs Non-BPCI difference	BPCI Hospital vs Non-BPCI difference	BPCI Both vs Non-BPCI difference
	% change				$ or percentage point change		
**Total episode spending, Mean $**	0.9	-2.4	-1.5	-3.0	-1,345	-1,010	-1,585
**90-day Readmissions, Mean %**	2.4	0.0	3.1	-2.0	-0.6	0.1	-1.1
**90-day Mortality, Mean %**	2.7	-4.8	-7.4	-7.5	-0.5	-0.7	-0.7
**Exploratory Outcomes**							
Spending							
Institutional PAC*	0.8	-6.5	-7.5	-9.5	-887	-1,062	-1,223
HHA	5.7	-36.7	11.8	-38.8	-331	52	-416
Utilization							
Discharge to institutional PAC Providers**, Mean %	1.4	-9.2	-6.8	-15.7	-5.5	-4.5	-9.0
Discharge with HHA, Mean %	4.8	-27.7	7.3	-30.5	-7.6	0.6	-9.4
SNF LOS, Mean days	0.2	-8.4	-6.6	-5.6	-1.4	-1.2	-0.9
HHA days, Mean days	3.4	-24.3	6.7	-31.5	-1.3	0.2	-1.9

Notes: BPCI=Bundled Payments for Care Improvement; ED=Emergency Department; HHA=Home Health Agency; LOS=Length of Stay; LTAC=Long-Term Acute Care; PAC=Post-Acute Care; SNF=Skilled Nursing Facility. *Combination of SNF, inpatient rehabilitation facility, and long-term acute care spending. **Discharge to skilled nursing facility or inpatient rehabilitation facility.

© 2022 Liao JM et al. *JAMA Health Forum.*

eFigure 6. Adjusted Changes in Surgical Episode Outcomes Associated with Bundled Payment Participation Among PGPs and Hospitals

Panel A. Total Episode Spending

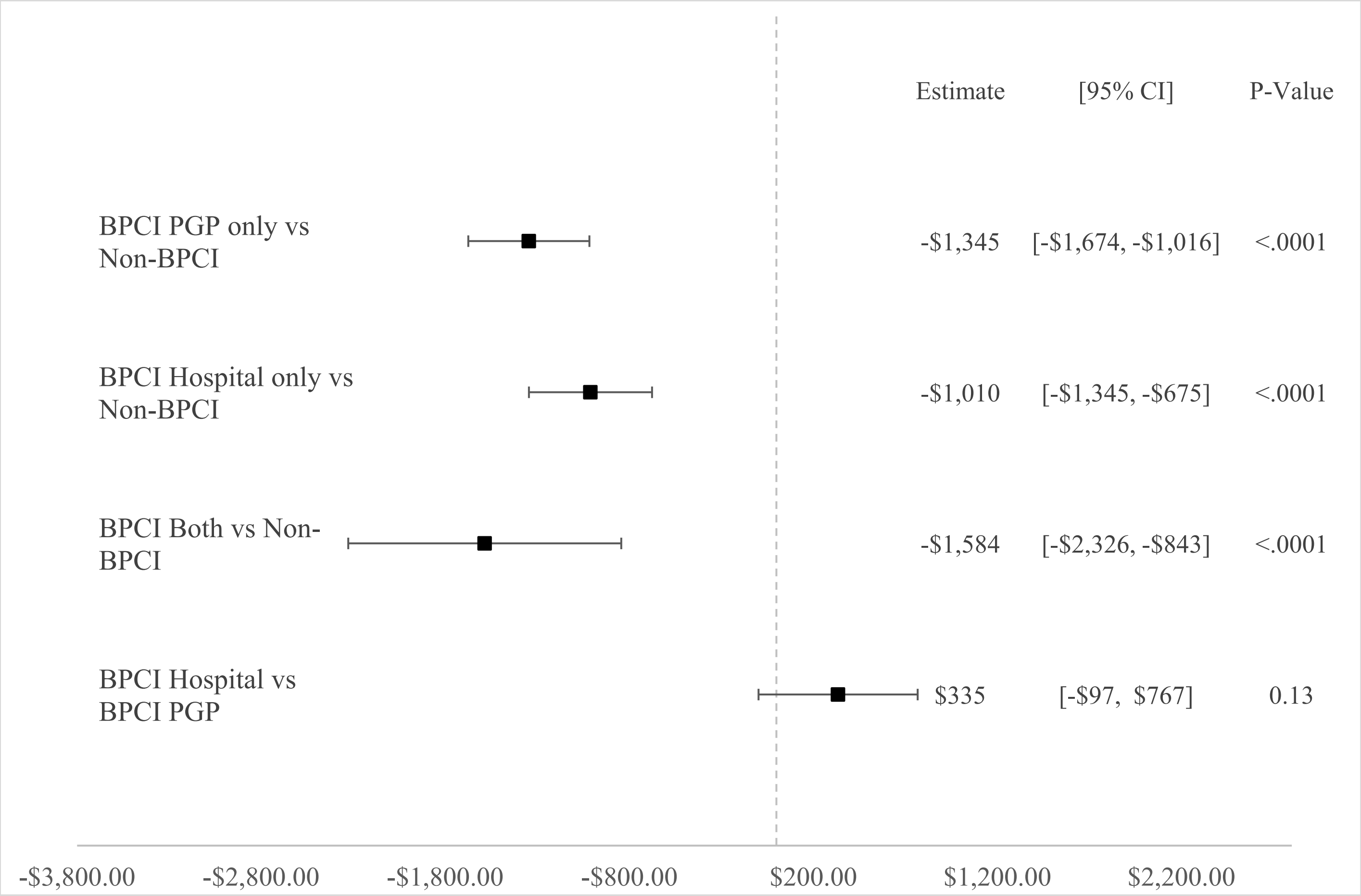

Notes: BPCI=Bundled Payments for Care Improvement; PGP=Physician Group Practice. Holm-Bonferroni correction was applied to the primary study outcome with corrected alpha as follows: BPCI Hospital vs Non-BPCI (p=0.0125; reject the null hypothesis), BPCI PGP vs Non-BPCI (p=0.0167; reject the null hypothesis), BPCI Both vs Non-BPCI (p=0.025; reject the null hypothesis), BPCI Hospital vs BPCI PGP (p=0.05; do not reject the null hypothesis).

© 2022 Liao JM et al. *JAMA Health Forum.*

Panel B. 90-day Readmissions and Mortality

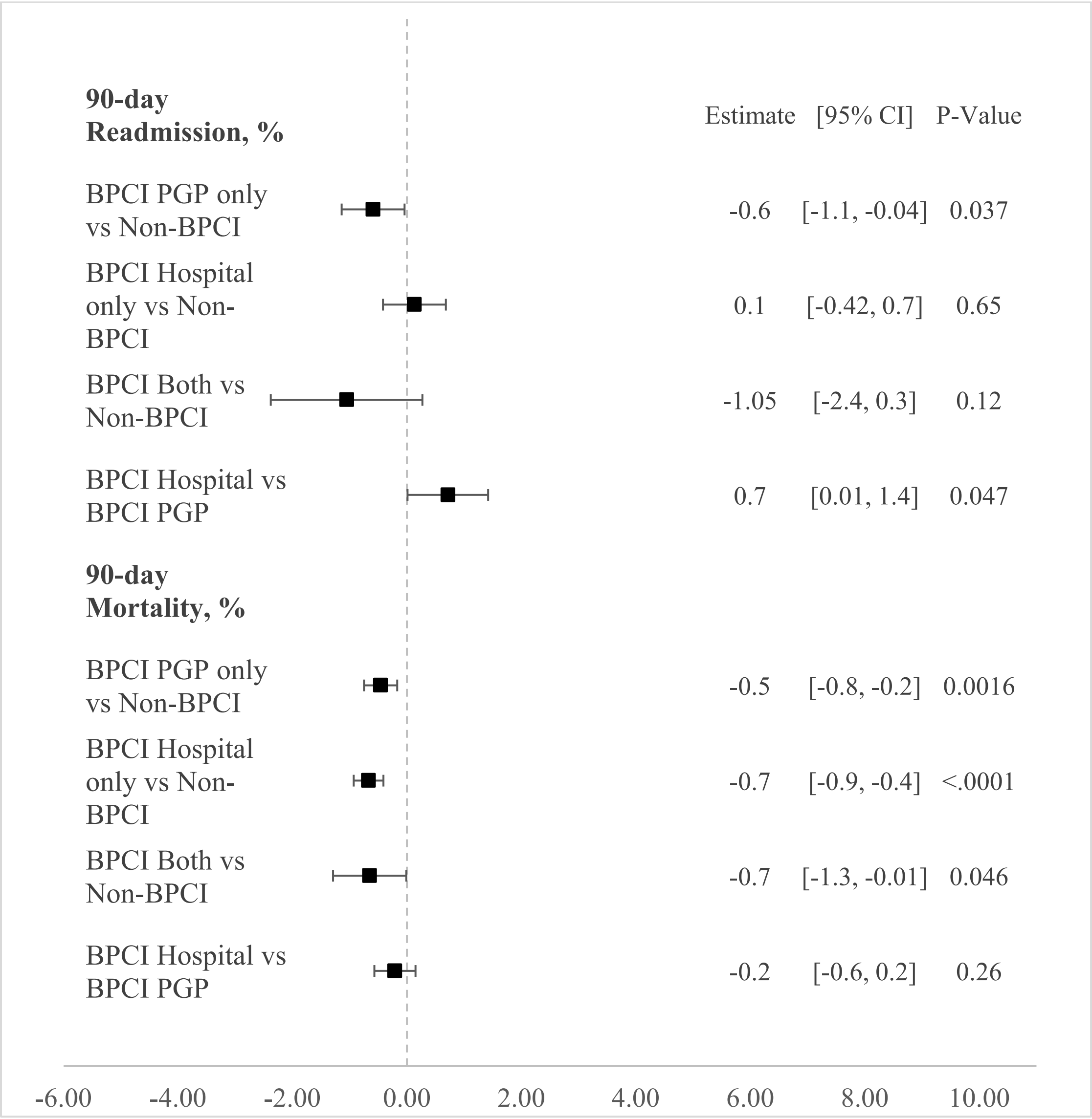

© 2022 Liao JM et al. *JAMA Health Forum.*

eTable 10. Adjusted Changes in Surgical Episode Exploratory Outcomes Associated With Bundled Payments

	BPCI PGP vs Non-BPCI	BPCI Hospital vs Non-BPCI	BPCI Both vs Non-BPCI	BPCI Hospital vs BPCI PGP
**Spending, $**				
Institutional PAC*	-1870***	-1063***	-1223***	-176
HHA	-331***	52	-416***	382***
**Utilization, percentage point**				
Discharge to Institutional PAC Providers**	-5.5***	-4.5***	-9.0***	1.0
Discharge with HHA	-7.6***	0.6	-9.4***	8.2***
SNF LOS	-1.4***	-1.2***	-0.9***	0.2
HHA days	-1.3***	0.2	-1.9***	1.4***

Notes: BPCI=Bundled Payments for Care Improvement; ED=Emergency Department; HHA=Home Health Agency; LOS=Length of Stay; LTAC=Long-Term Acute Care; PAC=Post-Acute Care; PGP=Physician Group Practice; SNF=Skilled Nursing Facility. *Combination of SNF, inpatient rehabilitation facility, and long-term acute care spending. **Discharge to skilled nursing facility or inpatient rehabilitation facility. ***$p<0.05$.

© 2022 Liao JM et al. *JAMA Health Forum.*

eFigure 7. Sensitivity Analysis for Changes in Total Episode Spending in Medical Episodes, Using Generalized Linear Models With Log Link and Gamma Distribution

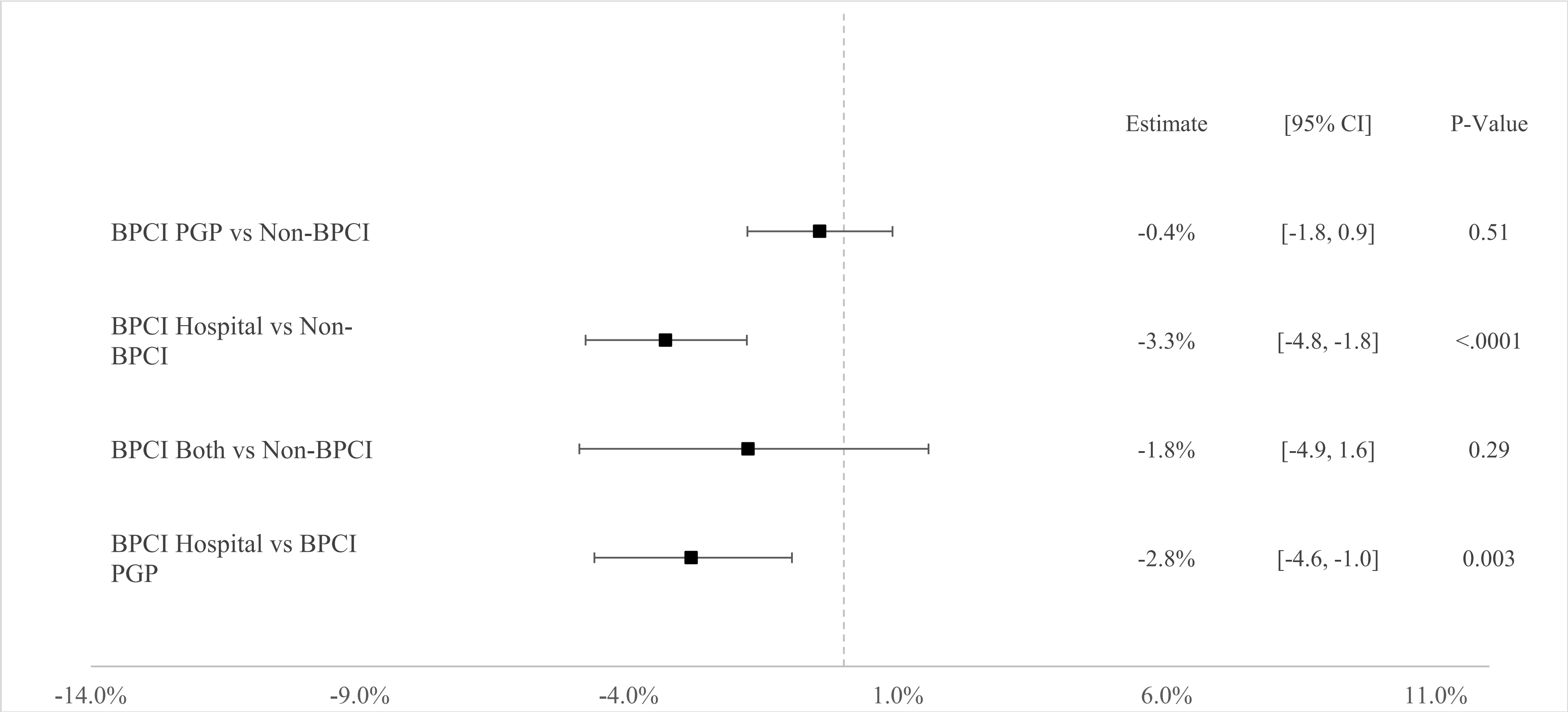

Notes: BPCI=Bundled Payments for Care Improvement; PGP=Physician Group Practice.

© 2022 Liao JM et al. *JAMA Health Forum.*

eFigure 8. Sensitivity Analysis for Changes in Total Episode Spending in Surgical Episodes, Using Generalized Linear Models With Log Link and Gamma Distribution

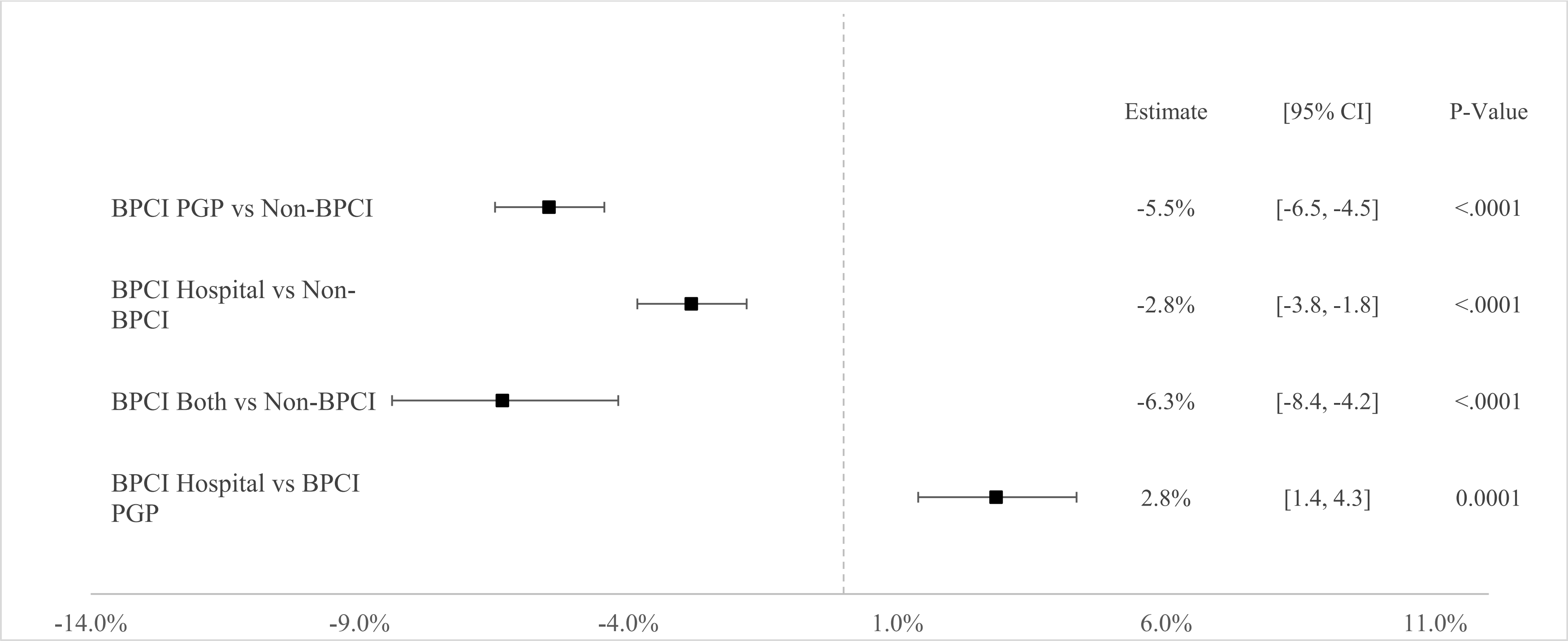

Notes: BPCI=Bundled Payments for Care Improvement; PGP=Physician Group Practice.

© 2022 Liao JM et al. *JAMA Health Forum.*

eFigure 9. Sensitivity Analysis for Changes in Total Episode Spending in Medical Episodes, Considering BPCI Both as BPCI PGP episodes

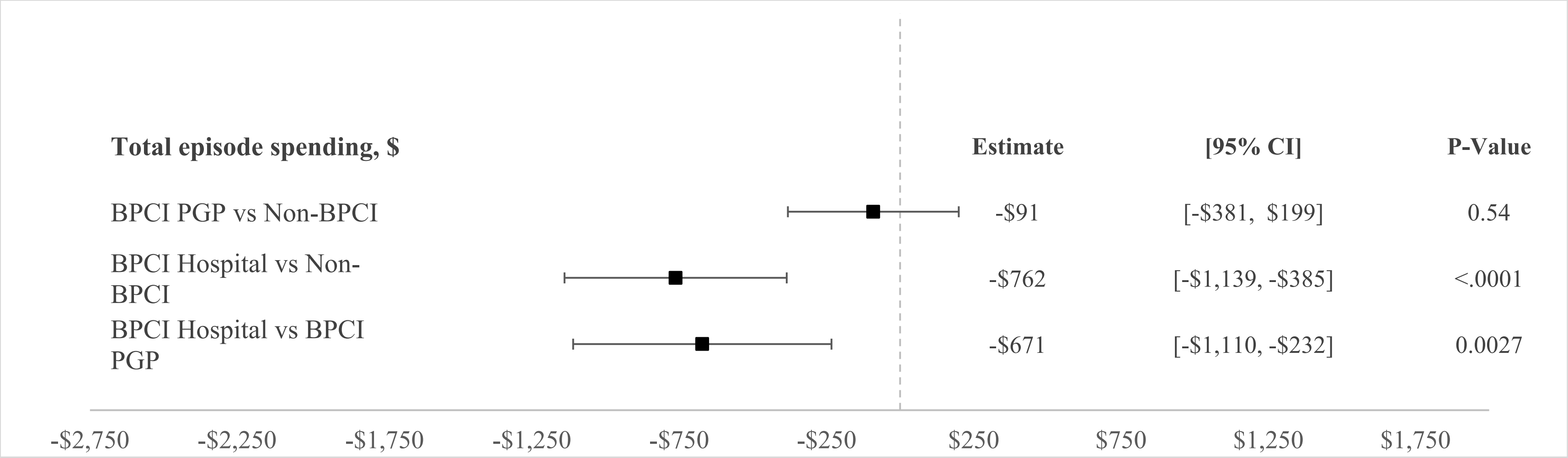

Notes: BPCI=Bundled Payments for Care Improvement; PGP=Physician Group Practice.

© 2022 Liao JM et al. *JAMA Health Forum.*

eFigure 10. Sensitivity Analysis for Changes in Total Episode Spending in Surgical Episodes, Considering BPCI Both as BPCI PGP episodes

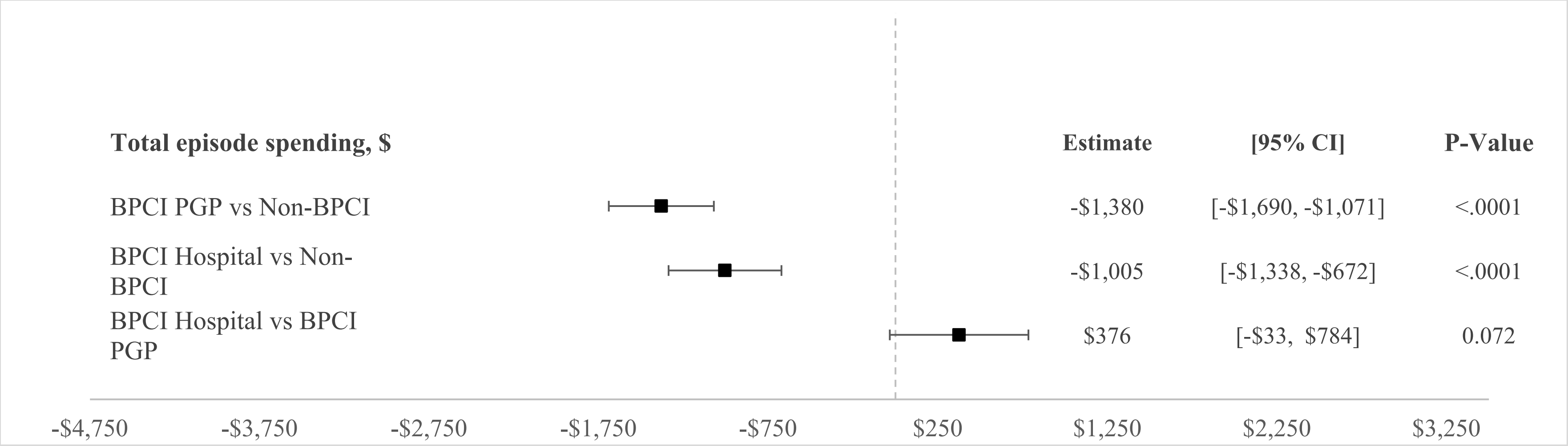

Notes: BPCI=Bundled Payments for Care Improvement; PGP=Physician Group Practice.

© 2022 Liao JM et al. *JAMA Health Forum.*

eFigure 11. Sensitivity Analysis for Changes in 90-day Mortality in Medical Episodes, Including Episodes with Death at Index Hospitalization

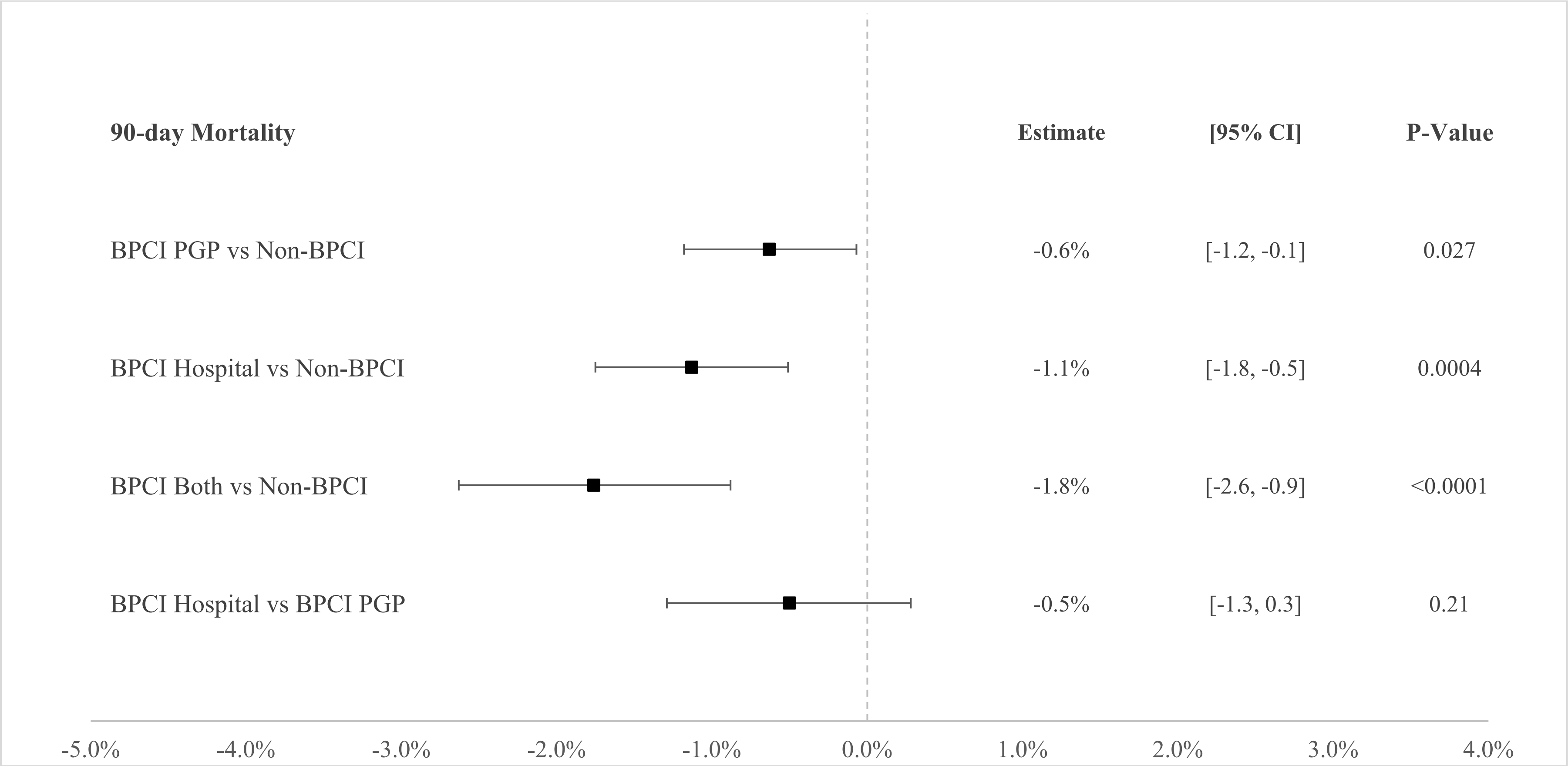

Notes: BPCI=Bundled Payments for Care Improvement; PGP=Physician Group Practice.

© 2022 Liao JM et al. *JAMA Health Forum.*

eFigure 12. Sensitivity Analysis for Changes in 90-day Mortality in Surgical Episodes, Including Episodes with Death at Index Hospitalization

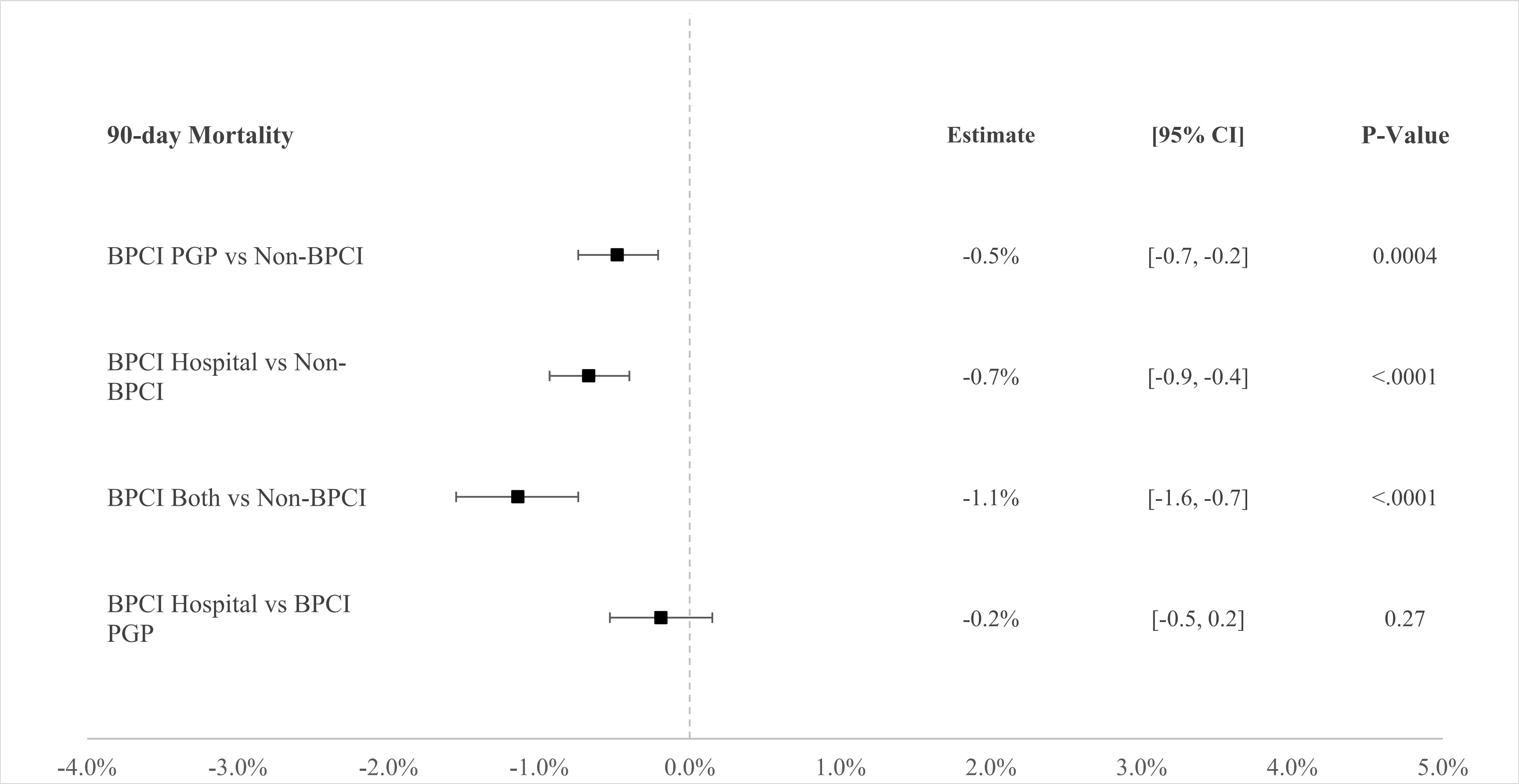

Notes: BPCI=Bundled Payments for Care Improvement; PGP=Physician Group Practice.

© 2022 Liao JM et al. *JAMA Health Forum.*

eMethods 4. Sensitivity Analysis Examining Robustness of the 90-day Mortality Outcome

1. Describing selection on observables - We plotted observable severity (Elixhauser score) by study group over time, which suggested selection based on observable characteristics. Our original model allowed for a time-varying relationship between Elixhauser score and mortality.

Medical episodes

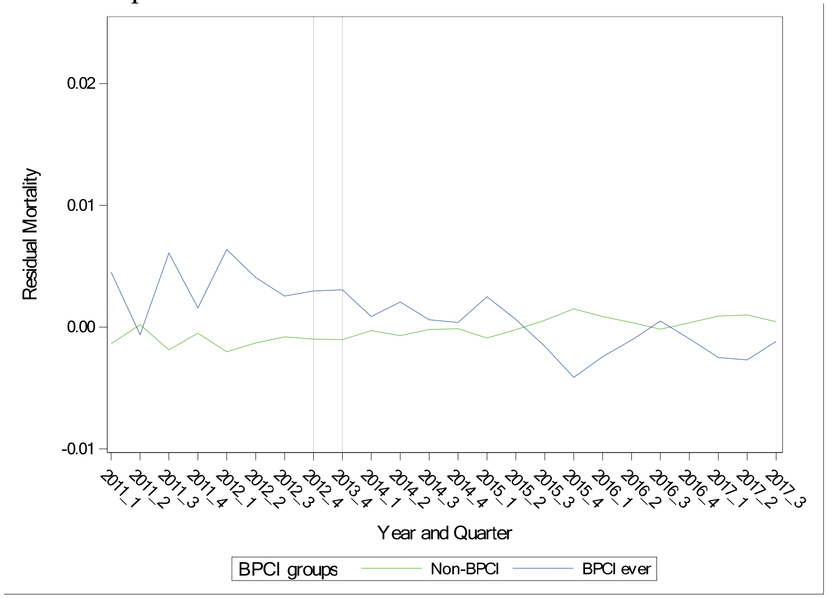

Surgical episodes

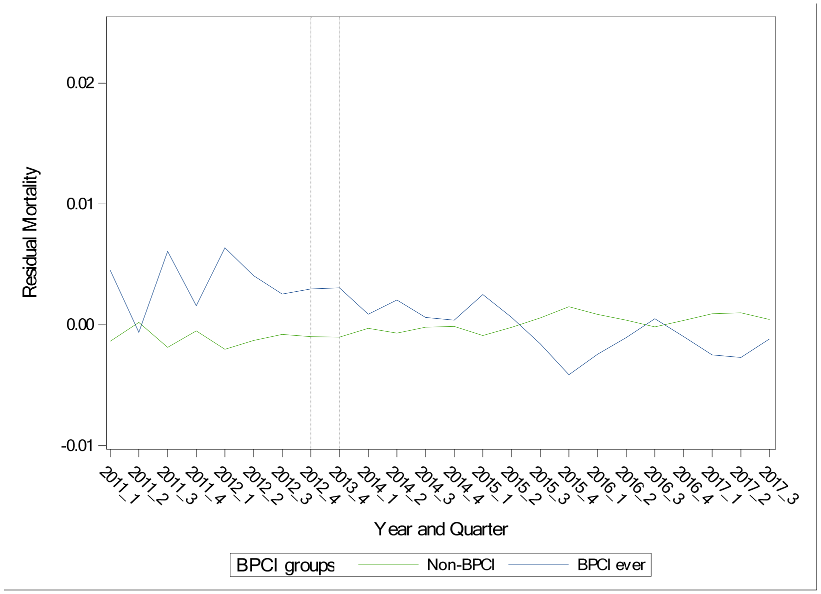

2. Describing selection on unobservables - We plotted residuals from the mortality model by study group (i.e., we regressed mortality on patient characteristics and then plotted the residuals averaged by BPCI vs. Non-BPCI group and quarter-year), which were suggestive of selection based on characteristics that were unobservable in our data. Note that residual mortality decreases for BPCI group (medical >> surgical) while the non-BPCI group is quite stable.

© 2022 Liao JM et al. *JAMA Health Forum.*

Medical episodes

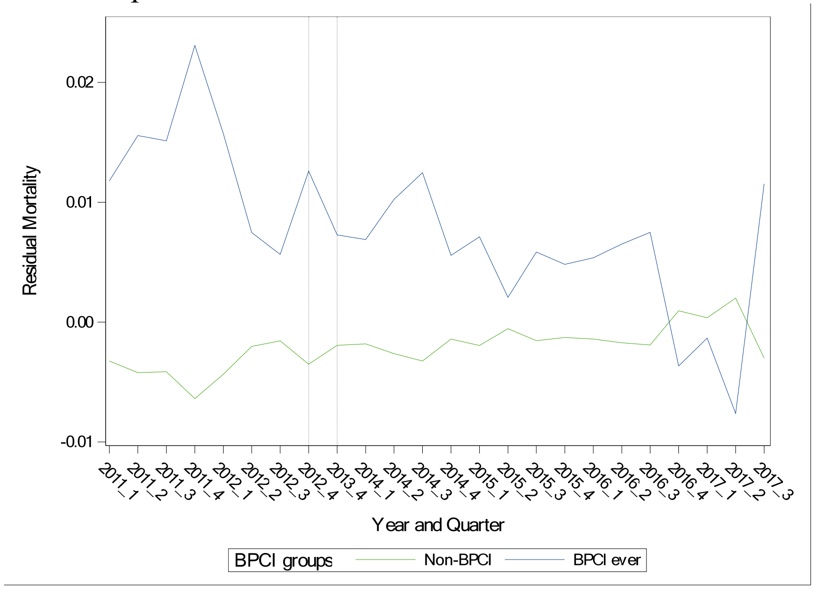

Surgical episodes

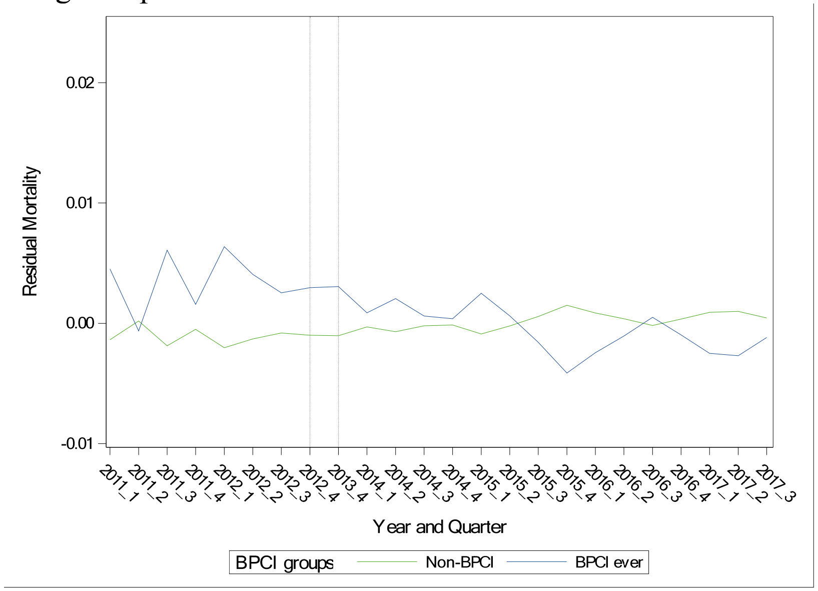

© 2022 Liao JM et al. *JAMA Health Forum.*

2. Mitigating selection on unobservables at area level - In an effort to account for selection at the area level (selection occurring based on where patients resided), we used data from the pre-intervention period and modeled observed mortality as a function of patient characteristics and episode type. This process generated mortality residuals (highly correlated with observed mortality), which were aggregated at the ZIP level and incorporated into our analyses.

Results from ordinary least squared models with versus without this zip-level mortality residual variable did not exhibit meaningful differences.

Medical episodes:

	Original OLS model without zip-level mortality residual			OLS model with zip-level mortality residual		
	Estimate	95% CI	P value	Estimate	95% CI	P value
BPCI PGPs vs. Non-BPCI	-0.67	(-1.25, -0.09)	0.0229	-0.61	(-1.19, -0.02)	0.0415
BPCI Hospitals vs. Non-BPCI	-1.14	(-1.76, -0.52)	0.0003	-1.24	(-1.82, -0.66)	<.0001
BPCI Both vs. Non-BPCI	-0.09	(-1.81, 1.63)	0.9208	-0.04	(-1.72, 1.65)	0.9672
BPCI Hospitals vs. BPCI PGPs	-0.46	(-1.27, 0.34)	0.2549	-0.63	(-1.41, 0.14)	0.1103

Surgical episodes:

	Original OLS model without zip-level mortality residual			OLS model with zip-level mortality residual		
	Estimate	95% CI	P value	Estimate	95% CI	P value
BPCI PGPs vs. Non-BPCI	-0.46	(-0.75, -0.17)	0.0016	-0.46	(-0.77, -0.16)	0.0026
BPCI Hospitals vs. Non-BPCI	-0.67	(-0.93, -0.41)	<.0001	-0.66	(-0.92, -0.4)	<.0001
BPCI Both vs. Non-BPCI	-0.65	(-1.29, -0.01)	0.0457	-0.62	(-1.4, 0.15)	0.1134
BPCI Hospitals vs. BPCI PGPs	-0.21	(-0.57, 0.15)	0.2608	-0.20	(-0.57, 0.18)	0.3012

Results from ordinary least squared models, with predicted values bounded between 0 and 1, with versus without this zip-level mortality residual variable also did not exhibit meaningful differences.

© 2022 Liao JM et al. *JAMA Health Forum.*

Medical episodes:

	Original OLS model without zip-level mortality residual			OLS model with zip-level mortality residual		
	Estimate	95% CI	P value	Estimate	95% CI	P value
BPCI PGPs vs. Non-BPCI	-0.67	(-1.25, -0.09)	0.0229	-0.61	(-1.19, -0.02)	0.0427
BPCI Hospitals vs. Non-BPCI	-1.14	(-1.76, -0.52)	0.0003	-1.24	(-1.82, -0.66)	<.0001
BPCI Both vs. Non-BPCI	-0.09	(-1.81, 1.63)	0.9208	-0.04	(-1.72, 1.65)	0.9675
BPCI Hospitals vs. BPCI PGPs	-0.46	(-1.27, 0.34)	0.2549	-0.64	(-1.41, 0.14)	0.1077

Surgical episodes:

	Original OLS model without zip-level mortality residual			OLS model with zip-level mortality residual		
	Estimate	95% CI	P value	Estimate	95% CI	P value
BPCI PGPs vs. Non-BPCI	-0.46	(-0.75, -0.17)	0.0016	-0.46	(-0.77, -0.16)	0.0026
BPCI Hospitals vs. Non-BPCI	-0.67	(-0.93, -0.41)	<.0001	-0.66	(-0.92, -0.4)	<.0001
BPCI Both vs. Non-BPCI	-0.65	(-1.29, -0.01)	0.0457	-0.62	(-1.4, 0.15)	0.1143
BPCI Hospitals vs. BPCI PGPs	-0.21	(-0.57, 0.15)	0.2608	-0.2	(-0.57, 0.17)	0.2999

Results from logistic regression sensitivity analysis with this zip-level mortality residual variable versus original OLS model also did not exhibit meaningful differences.

Medical episodes:

	Original OLS model without zip-level mortality residual			Logistic regressions model with zip-level mortality residual generated		
	Estimate	95% CI	P value	Estimate	95% CI	P value
BPCI PGPs vs. Non-BPCI	-0.67	(-1.25, -0.09)	0.0229	-0.61	(-1.19, -0.02)	0.0419
BPCI Hospitals vs. Non-BPCI	-1.14	(-1.76, -0.52)	0.0003	-1.24	(-1.82, -0.66)	<.0001
BPCI Both vs. Non-BPCI	-0.09	(-1.81, 1.63)	0.9208	-0.04	(-1.73, 1.64)	0.9603
BPCI Hospitals vs. BPCI PGPs	-0.46	(-1.27, 0.34)	0.2549	-0.63	(-1.41, 0.15)	0.1126

© 2022 Liao JM et al. *JAMA Health Forum.*

Surgical episodes:

	Original OLS model without zip-level mortality residual			OLS model with zip-level mortality residual generated from a logistic regression		
	Estimate	95% CI	P value	Estimate	95% CI	P value
BPCI PGPs vs. Non-BPCI	-0.46	(-0.75, -0.17)	0.0016	-0.47	(-0.77, -0.17)	0.0024
BPCI Hospitals vs. Non-BPCI	-0.67	(-0.93, -0.41)	<.0001	-0.66	(-0.92, -0.4)	<.0001
BPCI Both vs. Non-BPCI	-0.65	(-1.29, -0.01)	0.0457	-0.62	(-1.4, 0.15)	0.1151
BPCI Hospitals vs. BPCI PGPs	-0.21	(-0.57, 0.15)	0.2608	-0.19	(-0.56, 0.18)	0.3067

3. In an effort to account for selection at the patient level (selection occurring based on unobserved characteristics of individuals), we used a similar approach from step #2 (data from the pre-intervention period, modeling observed mortality as a function of patient characteristics and episode type) to generate mortality residuals. We then modeled mortality residuals as a function of additional patient and market characteristics to generate predicted values for mortality residuals. These predicted values were then incorporated into our analyses. Results from these models with versus without this individual-level predicted mortality residual variable also did not exhibit meaningful differences from our original results.

Medical episodes:

	Original OLS model without individual-level mortality residual			OLS model with individual-level mortality residual		
	Estimate	95% CI	P value	Estimate	95% CI	P value
BPCI PGPs vs. Non-BPCI	-0.67	(-1.25, -0.09)	0.0229	-0.67	(-1.25, -0.08)	0.0256
BPCI Hospitals vs. Non-BPCI	-1.14	(-1.76, -0.52)	0.0003	-1.17	(-1.75, -0.59)	<.0001
BPCI Both vs. Non-BPCI	-0.09	(-1.81, 1.63)	0.9208	-0.12	(-1.81, 1.57)	0.8892
BPCI Hospitals vs. BPCI PGPs	-0.46	(-1.27, 0.34)	0.2549	-0.5	(-1.28, 0.27)	0.2049

© 2022 Liao JM et al. *JAMA Health Forum.*

Surgical episodes:

	Original OLS model without individual-level mortality residual			OLS model with individual-level mortality residual		
	Estimate	95% CI	P value	Estimate	95% CI	P value
BPCI PGPs vs. Non-BPCI	-0.46	(-0.75, -0.17)	0.0016	-0.46	(-0.76, -0.16)	0.0026
BPCI Hospitals vs. Non-BPCI	-0.67	(-0.93, -0.41)	<.0001	-0.67	(-0.93, -0.41)	<.0001
BPCI Both vs. Non-BPCI	-0.65	(-1.29, -0.01)	0.0457	-0.65	(-1.42, 0.12)	0.1000
BPCI Hospitals vs. BPCI PGPs	-0.21	(-0.57, 0.15)	0.2608	-0.21	(-0.58, 0.16)	0.2683

© 2022 Liao JM et al. *JAMA Health Forum.*

Collectively, these steps demonstrated that our findings were robust to inclusion of the zip-level mortality residual variable, as well as individual-level predicted mortality residuals (we also replicated results for episode spending using these approaches, which did not affect estimates of hospital or PGP participation [data not shown]). While the residuals in Step 2 were highly correlated with observed mortality, the predicted residuals in Step 3 were not well correlated with observed mortality (see below). Thus, our Step 3 analyses must be interpreted with caution.

	Scenario 1: use OLS for Step #1			Scenario 2: use OLS with predicted value bounded between 0 and 1 for Step #1			Scenario 3: use Logistic Regression for Step #1		
	Mortality	Mortality residuals	Predicted mortality residuals	Mortality	Mortality residuals	Predicted mortality residuals	Mortality	Mortality residuals	Predicted mortality residuals
Mortality									
Correlation	1	0.95	0.04	1	0.96	0.23	1	0.94	0.039
p-value		<.0001	<.0001		<.0001	<.0001		<.0001	<.0001
Mortality residuals from Step #1									
Correlation	0.95	1	0.042	0.96	1	0.056	0.94	1	0.047
p-value	<.0001		<.0001	<.0001		<.0001	<.0001		<.0001
Predicted mortality residuals from Step #2									
Correlation	0.04	0.042	1	0.23	0.056	1	0.039	0.047	1
p-value	<.0001	<.0001		<.0001	<.0001		<.0001	<.0001	

© 2022 Liao JM et al. *JAMA Health Forum.*

References

1. Gu XS, Rosenbaum PR. Comparison of Multivariate Matching Methods: Structures, Distances, and Algorithms. *Journal of Computational and Graphical Statistics* 1993;2(4):405-20. doi: 10.1080/10618600.1993.10474623
2. Zeldow B, Hatfield LA. Confounding and Regression Adjustment in Difference-in-Differences. 2019. https://ui.adsabs.harvard.edu/abs/2019arXiv191112185Z (accessed November 01, 2019).
3. Navathe AS, Liao, J.M., Linn, K.A., Zhang, Y., Mishra, A., Wang, R., Dinh, C.T., Zhu, J., Cousins, D.S., Lindner, J. and Emanuel, E.J. Spillover Effects of Medicare's Voluntary Bundled Payments for Joint Replacement Surgery to Patients Insured by Commercial Health Plans. *Annals of Internal Medicine* 2021;174(2):200-08. doi: 10.7326/m19-3792 %m 33347769
4. Zeldow B, Hatfield LA. Confounding and regression adjustment in difference-in-differences studies. *Health Services Research* 2021;56(5):932-41. doi: https://doi.org/10.1111/1475-6773.13666
5. Rolnick JA, Liao JM, Emanuel EJ, et al. Spending and quality after three years of Medicare's bundled payments for medical conditions: quasi-experimental difference-in-differences study. *BMJ* 2020;369:m1780. doi: 10.1136/bmj.m1780

© 2022 Liao JM et al. *JAMA Health Forum.*
