# Supplementary material for: Performance of Physician Groups and Hospitals Participating in Bundled Payments Among Medicare Beneficiaries
Source: JAMA Health Forum. 2022 Dec 29;3(12):e224889. doi: 10.1001/jamahealthforum.2022.4889 (PMC9856773; doi:10.1001/jamahealthforum.2022.4889)
Supplement: Supplement 2. — Data Sharing Statement [file jamahealthforum-e224889-s002.pdf]

Data Sharing Statement

Liao. Performance of Physician Groups and Hospitals Participating in Bundled Payments Among Medicare Beneficiaries. *JAMA Health Forum*. Published December 29, 2022. doi:10.1001/jamahealthforum.2022.4889

Data
Data available: No

Additional Information
Explanation for why data not available: Data is accessed in Medicare's virtual research data center, which precludes removal of any data below cell size of 11.
